# Prediction of heart failure risk factors from retinal optical imaging via explainable machine learning

**DOI:** 10.3389/fmed.2025.1551557

**Published:** 2025-03-17

**Authors:** Sona M. Al Younis, Samit Kumar Ghosh, Hina Raja, Feryal A. Alskafi, Siamak Yousefi, Ahsan H. Khandoker

**Affiliations:** ^1^Department of Biomedical Engineering and Biotechnology, Healthcare Engineering Innovation Group (HEIG), Khalifa University, Abu Dhabi, United Arab Emirates; ^2^Department of Mathematics and Computer Science, Fisk University, Nashville, TN, United States; ^3^Department of Genetics, Genomics, and Informatics, University of Tennessee Health Science Center, Memphis, TN, United States

**Keywords:** cardiovascular diseases, heart failure, machine learning, deep learning, explainable AI, UK Biobank

## Abstract

Over 64 million people worldwide are affected by heart failure (HF), a condition that significantly raises mortality and medical expenses. In this study, we explore the potential of retinal optical coherence tomography (OCT) features as non-invasive biomarkers for the classification of heart failure subtypes: left ventricular heart failure (LVHF), congestive heart failure (CHF), and unspecified heart failure (UHF). By analyzing retinal measurements from the left eye, right eye, and both eyes, we aim to investigate the relationship between ocular indicators and heart failure using machine learning (ML) techniques. We conducted nine classification experiments to compare normal individuals against LVHF, CHF, and UHF patients, using retinal OCT features from each eye separately and in combination. Our analysis revealed that retinal thickness metrics, particularly ISOS-RPE and macular thickness in various regions, were significantly reduced in heart failure patients. Logistic regression, CatBoost, and XGBoost models demonstrated robust performance, with notable accuracy and area under the curve (AUC) scores, especially in classifying CHF and UHF. Feature importance analysis highlighted key retinal parameters, such as inner segment-outer segment to retinal pigment epithelium (ISOS-RPE) and inner nuclear layer to the external limiting membrane (INL-ELM) thickness, as crucial indicators for heart failure detection. The integration of explainable artificial intelligence further enhanced model interpretability, shedding light on the biological mechanisms linking retinal changes to heart failure pathology. Our findings suggest that retinal OCT features, particularly when derived from both eyes, have significant potential as non-invasive tools for early detection and classification of heart failure. These insights may aid in developing wearable, portable diagnostic systems, providing scalable solutions for personalized healthcare, and improving clinical outcomes for heart failure patients.

## 1 Introduction

Heart failure (HF) is a significant global health condition that threatens more than 64 million individuals globally. It is a major contributor to healthcare expenses and the main reason for hospital admissions, especially for elderly individuals (1). In many countries, heart failure is the primary cause of cardiovascular-related deaths and disability-adjusted life years (2). A coordinated strategy centered on early detection, efficient treatment, and public health programs aiming at risk factor management is required to address this global epidemic. The classification of HF into distinct types allows for more targeted treatment strategies (3, 4). In this study, we focus on three main types: left ventricular heart failure (LVHF), congestive heart failure (CHF), and unspecified heart failure (UHF).

The most frequent type of HF is LVHF, often known as left-sided heart failure (5). It is caused by a weakening or stiffening of the left ventricle, the heart’s main pumping chamber. There are two other subtypes of LVHF: systolic heart failure (HFrEF) and diastolic heart failure (HFpEF). HFrEF is characterized by a reduced heart’s capacity to contract, which lowers the heart’s ejection fraction (EF); damage from a heart attack or persistent hypertension are common causes of this kind of heart failure. Patients with HFpEF have a normal ejection fraction, but a stiffened left ventricle that restricts blood flow. The failure of the ventricle to relax effectively during diastole causes fluid to accumulate in the lungs and tissues in this illness, which is usually linked to advanced age, obesity, and hypertension. However, CHF is a clinical term that describes a condition where fluid buildup becomes prominent throughout the body due to impaired heart function. CHF can affect the left or right side of the heart, but it is most commonly associated with biventricular heart failure, where both sides of the heart are compromised (6). The heart’s reduced pumping efficiency in CHF leads to congestion, or fluid accumulation, in various tissues, particularly the lungs, liver, and lower extremities. UHF is a broad classification used when a patient presents with symptoms of heart failure, but the specific type—whether systolic, diastolic, left-sided, or right-sided—has not been identified. This category is often applied during the initial stages of diagnosis when further testing is necessary to determine the precise nature of the heart failure. It may also be used in cases where heart failure coexists with other complicating conditions, making it challenging to categorize heart failure into well-defined types (7, 8).

The retina, an anterior extension of the central nervous system, exhibits notable anatomical and physiological similarities to the brain. Furthermore, retinal vasculature is the only vascular tissue that can be directly observed, and it exhibits embryological, physiological, and anatomical similarities to the coronary vasculature (9). Alterations in the retinal microvasculature may indicate chronic vascular damage due to multiple cardiovascular risk factors, including aging, diabetes mellitus, hypertension, and conditions such as vascular dementia. This may be because the microcirculation is responsible for determining the necessary adjustments in vascular tone to meet the oxygen demands of local tissues (10). The endothelial release of compounds, such as nitric oxide, other reactive oxygen species, and arachidonic acid metabolites, also contributes to the modulation of vascular tone in the microvasculature. Therefore, microvascular dysfunction is a potent indicator of cardiovascular events and may serve as a valuable target for developing novel treatment and prevention strategies. A chronic inflammatory condition affecting the vasculature throughout the body is atherosclerosis, a prevalent feature of cardiovascular diseases (CVD). Furthermore, retinal vascular geometry has been demonstrated to be associated with cardiac function, and endothelial dysfunction and microvascular disease also play significant roles in the development and progression of cardiovascular diseases, including heart failure.

Moreover, diabetic retinopathy has been linked to an elevated risk of CVD, peripheral neuropathy, renal disease, and mortality for decades, as evidenced by extensive research (11–14). Although the predictive relationship remains unclear, individuals with CVD are known to have an elevated risk of developing retinal vascular occlusions (15). The development of hypertension was associated with retinal arteriolar narrowing and retinal venular widening, independent risk factors (16, 17).

Researchers have examined associations between age, blood pressure, arterial stiffness index, body mass index (BMI), and retinal vasculometry. This procedure measures the diameter of the arteriolar and venular blood vessels and arterial stiffness. The retinal arteriolar diameter decreases as age increases, while the retinal venular diameter increases. Both diameters decrease as systolic blood pressure increases. It has been reported that these vascular alterations are direct indicators of the risk of stroke and other cardiovascular diseases (18). These findings suggest that the eye may provide valuable insights into heart health—assuming no eye-specific diseases are present—as changes in retinal vasculature can often reflect systemic vascular aging or underlying conditions (19). Flicker light-induced dilatation of retinal arterioles and venules, along with non-invasive analyses of retinal vessel diameters, have proven to be sensitive and reliable diagnostic tools for cardiovascular risk stratification and treatment strategy monitoring in both primary and secondary prevention across younger or older populations with or without CVD (20).

The deep learning (DL) models and advanced image analysis techniques can extract meaningful features (such as vessel caliber, vessel geometry, and retinal layers) from the ocular images [fundus images, optical coherence tomography (OCT), and optical coherence tomography angiography (OCT-A)] which can be passed to the prediction models (19). As summarized in Table 1, the study (21) investigated the potential of fundus images to predict cardiometabolic risk factors, including age, sex, blood pressure, smoking status, glycaemic status, total lipid panel, sex steroid hormones, and bioimpedance measurements. It was concluded that the retina stores unique information about blood pressure, hemoglobin A1c, relative fat mass, and sex, and a fundus image can reliably predict age and sex. Hamada et al. (22) employed a novel multi-modal approach based on DL techniques for distinguishing the CVD group from the control group, which involved designing a case–control study that combined data from multiple modalities—including DXA and retinal images. It was concluded that the proposed model can recognize and implement specific prognostic indicators for ischemic heart disease and hypertension. Ehsan et al. (23) developed a DL model that could tell from retinal images and limited demographic information if a person has a high-risk score for ASCVD. The retinal images, age, race/ethnicity, and sex at birth were combined to create the DL model, designed to predict an individual’s 10-year ASCVD risk score. The pooled cohort equation (PCE) provided the ground truth. Subsequently, the model was evaluated on the US EyePACS 10 K dataset, which consisted of 18,900 images from 8,969 diabetic individuals and comprised 5.8% non-Hispanic White and 99.9% diabetic population. A PCE score of ≥7.5% was used to define an elevated risk of ASCVD. The study (24) presented a new approach to detecting CVD by analyzing retinal fundus images. The principal process involved the extraction of tissue from retinal vessels to diagnose and treat CVD. The Gaussian filter was applied to retinal images, followed by binarization, circle fitting, and statistical data extraction to identify the optic disc. The Chronological Chef Based Optimisation Algorithm (CCBOA)-based Res-Unet separates the blood vessels. In the study (25), we introduce a novel SSL-based foundation model for retinal images (RETFound) and comprehensively assess its generalizability and efficacy in various disease detection tasks. A large AI model trained on a vast quantity of unlabelled data at scale is called a foundation model. This model can be adapted to a broad range of downstream tasks. They employed an advanced SSL technique (masked autoencoder) to create two distinct RETFound models, one utilizing retinal images and the other utilizing OCT. They evaluated the model performance in predicting systemic diseases such as heart failure, ischaemic stroke, myocardial infarction, and Parkinson’s disease with retinal images. RETFound has demonstrated substantial improvement in internal evaluations for CFP and OCT, even though the overall performance was compromised in these complex tasks. The study’s objective (26) was to analyze the potential differences in retinal microvascular and structural parameters (i.e., capillary vessel density and retinal layer thickness) between patients with HFpEF and control individuals as determined by OCTA. They evaluated the correlations between retinal parameters and clinical and echocardiographic parameters in HFpEF. Only patients with HFpEF underwent echocardiography, while controls did not. Volume scans of both eyes were computed as 6 × 6 mm volumes centered on the macula. The study (27) reported that OCT is crucial in diagnosing cardiovascular diseases and identifying plaques. Usually, physicians manually analyze OCT images to identify vulnerable plaques, a process prone to subjective errors and high workload. To address this, a convolutional neural network (CNN)-based model was developed to improve diagnostic accuracy and efficiency. The model learned multilevel features from raw OCT images and employed decision-making layers to classify and recognize vulnerable plaques. Experimental results using clinically labeled datasets showed that the CNN model achieved a high recognition rate, offering significant support for early diagnosis, intervention, and prevention of cardiovascular diseases.

**Table 1:** Summarizing the studies detecting CVD through ocular imaging.

Studies	Year	Cohort	Modality	Model	Results
Gerrits et al. (21)	2020	Qatar Biobank	Fundus	MobileNet-V2	Age: MAE: 2.78 years Systolic blood pressure: MAE: 8.96 mmHg Diastolic blood pressure MAE: 6.84 mmHg Hemoglobin A1c: MAE: 0.61% Relative fat mass: MAE: 5.68 units Testosterone: MAE: 3.76 nmol/L Sex: AUC:0.97
Al-Absi et al. (22)	2022	Qatar Biobank	Retinal images Dual-energy X-ray absorptiometry (DXA)	Machine learning models and DL (ResNet-34)	Accuracy retinal images: 75.6% DXA: 77.4% Multi-modal: 78.3%
Vaghefi et al. (23)	2024	UK Biobank, and EyePACS 10 K	Retinal images, Limited demographic data	DL (Resnet-V2/ResNet50)	UK-Biobank AUC:0.89 Sensitivity: 84% Specificity: 90%
Kadry et al. (24)	2024	JSIEC	Fundus Images	Chronological Chef Based Optimisation Algorithm (CCBOA) and Deep Residual Network (DRN).	Accuracy: 89.8% NPV: 86.4% PPV: 86.8% TNR: 90.5% TPR: at 90.1%
Zhou et al. (25)	2023	MEH-AlzEye UK-biobank	Fundus, OCT	ViT	AUROC: UK Biobank OCT Myocardial infarction: 0.605 (0.59, 0.621) Ischaemic stroke: 0.559 (0.541, 0.577) Heart failure: 0.682 (0.678, 0.685) Parkinson’s disease: 0.551 (0.534, 0.567)
Weerts et al. (26)	2024	Local dataset from Maastricht University	Echocardiographic parameters OCTA	Statistical model	*p* = 0.027 (74 [68–80] vs. 68 [58–77] years) *p* = 0.034 (73% vs. 42% females)

Retinal layers, including the ISOS-RPE (inner segment/outer segment to retinal pigment epithelium), INL-ELM (inner nuclear layer to external limiting membrane), ELM-ISOS (external limiting membrane to inner segment/outer segment junction), and INL-RPE (inner nuclear layer to retinal pigment epithelium) may provide important information about the vascular and structural integrity of the eye. They may indicate systemic conditions, such as cardiovascular health (28, 29). Research has demonstrated that the retinal vasculature is comparable to the cerebral and coronary circulatory systems, suggesting it may be used as a biomarker for heart failure and other cardiovascular disorders (22). Retinal thickness variations, particularly in macular subfields, may indicate microvascular disease (30–32).

Despite advances in heart failure diagnosis and management, the intricate relationship between retinal measurements and HF types remains insufficiently understood. Providing a non-invasive, easily accessible approach to aid in diagnosing these different heart failure types without the need for costly or highly specialized equipment would significantly enhance patient care. Machine learning (ML) techniques, including deep learning, hold promise for leveraging retinal measurements to predict the specific type of heart failure.

This study investigates how well retinal optical characteristics, such as retinal layer and macular thickness measurements, can predict heart failure types. We aim to explore the relationship between these ocular indicators and heart failure using Machine Learning models trained on retinal data. This innovative strategy emphasizes retinal health as a possible proxy for cardiovascular function, providing a widely available, non-invasive diagnostic tool that may supplement conventional techniques. This study will also evaluate whether retinal measurements from the right eye, left eye, or a combination of both, provide greater predictive accuracy in detecting HF. By systematically comparing these ocular measurements, we aim to uncover novel insights into the systemic effects of the heart. Additionally, we will incorporate explainable AI techniques to understand better the importance of individual retinal features and their correlations with specific heart failure types. This approach will enhance the interpretability of our machine-learning models and shed light on the underlying biological mechanisms linking retinal changes to heart failure pathology.

## 2 Methods

### 2.1 Data collection

The UK Biobank, established between 2006 and 2010, is a large-scale prospective cohort study and a comprehensive biomedical research resource. It includes data from 502,000 adults aged 40–69 across England, Scotland, and Wales. The database encompasses an extensive array of genetic, physiological, and lifestyle information, with participants’ health outcomes systematically tracked through electronic health records (33, 34). Participants contributed to this resource by attending a baseline assessment visit after informed consent. During this visit, detailed information on their health, lifestyles, hearing, and cognitive function was collected through a touchscreen questionnaire and a brief verbal interview. Various physical measurements were also performed, including assessments of blood pressure, arterial stiffness, eye health, body composition, handgrip strength, ultrasound bone densitometry, spirometry, and fitness testing with electrocardiography. Furthermore, biological samples such as blood, urine, and saliva were obtained for subsequent analyses, enhancing the depth of the dataset. The present analysis of the UK Biobank data was conducted and approved by the North West Multi-Centre Research Ethics Committee (reference no. 06/MRE08/65). The study followed the guidelines outlined in the Declaration of Helsinki, and all participants provided written informed consent. Detailed information about the survey can be found on the UK Biobank website.^1^

The study initially included a cohort of 57,636 participants. Health-related outcomes in the UK Biobank were identified using the International Classification of Diseases version 10 (ICD-10) codes, specifically code I50.0 for congestive heart failure, I50.1 for left ventricular failure, and code I50.9 for unspecified heart failure, extracted from hospital records (data field ID 41270, UK Biobank dataset). The prevalence of these conditions was distributed as follows: 8,858 cases of congestive heart failure, 10,380 cases of left ventricular failure, 8,397 cases of unspecified heart failure, and 2,922 normal cases (participants not having ICD-10). However, participants missing information for left and right eye images were excluded from further analysis. Consequently, the dataset comprised a total of 2,824 patients, categorized into 701 Normal (Type 0), 744 Left Ventricle Heart Failure (Type 1), 701 Congestive Heart Failure (Type 2), and 678 Unspecified Heart Failure (Type 3).

### 2.2 Ophthalmic assessments

Ophthalmic assessments were conducted for a subset of participants between 2009 and 2010 at six assessment centers. These assessments included visual acuity (LogMAR) measurements, refractive error, intraocular pressure (IOP), and ophthalmic imaging captures. Baseline best-corrected visual acuity was measured using a computerized semi-automated system at a distance of 3 meters. Autorefraction was performed using an RC5000 Auto Refkeratometer (Tomey, Nagoya, Japan), and the spherical equivalent was calculated by summing the spherical power and half of the cylindrical power. Corneal compensated intraocular pressure (IOPcc) was measured with the Ocular Response Analyzer (ORA; Reichert Corp., Philadelphia, PA), with one measurement taken per eye. Participants with possible eye infections or recent eye surgery (within 4 weeks) were excluded from IOP measurements. Single-field color fundus photographs (45° field-of-view, centered on the optic disc and macula and including both) and macular OCT scans were captured using a digital Topcon-1000 integrated ophthalmic camera (Topcon 3D OCT1000 Mark II, Topcon Corp., Tokyo, Japan) (9).

### 2.3 Spectral-domain optical coherence tomography imaging protocol

The Topcon 3D OCT 1000 Mk2 (Topcon Corp., Tokyo, Japan) was used for Spectral-domain OCT imaging. This was done after collecting visual acuity, autorefraction, and intraocular pressure (IOP) measurements. The OCT images were captured under mesopic lighting conditions without pupillary dilation, utilizing the 3-dimensional 6 × 6 mm macular volume scan mode, which includes 512 A-scans per B-scan and 128 horizontal B-scans in a raster pattern. The right eye was photographed first, followed by the left (35).

### 2.4 Data processing

The patient cohort was categorized into four groups based on heart failure type: Normal, Left Ventricular Heart Failure, Congestive Heart Failure, and Unspecified Heart Failure. The statistical analysis involved computing each parameter’s means, standard deviations, and counts. Data processing and analysis were performed using Python, utilizing **pandas** for data management, **scipy** for statistical testing, and **seaborn** and **Matplotlib** for generating visualizations such as box plots. Pairwise t-tests were employed to analyze differences in eye measurements across the heart failure types. Each retinal measurement was compared against the baseline group (Type 0: Normal) to assess significant variations in the other groups (Types 1, 2, and 3). A *p*-value of less than 0.05 was considered statistically significant, indicating that observed differences in eye measurements between ‘Normal’ patients and those with specific types of heart failure were unlikely to be due to random chance. Additionally, box plots were generated for each retinal measurement to visually illustrate the distribution of data points across the heart failure categories. The dataset includes optical coherence tomography (OCT) imaging data for both left and right eyes, offering a comprehensive view of retinal features. Detailed descriptions and definitions of these retinal optical features are provided in Supplementary Table S1.

### 2.5 Experiment setup

A set of extensive tests was carried out to investigate the influence of different retinal OCT features on the classification of heart failure. Nine configurations were used to arrange the trials, with classifications for measurements taken with the left, right, and both eyes. These setups involved differentiating between normal and heart failure types: LVHF, CHF, and UHF. To be more precise, we classified the following: (1) Normal versus LVHF using left eye measurements; (2) Normal versus CHF using left eye measurements; (3) Normal versus UHF using left eye measurements; (4) Normal versus LVHF using right eye measurements; (5) Normal versus CHF using right eye measurements; (6) Normal versus UHF using right eye measurements; (7) Normal versus LVHF using both eyes; (8) Normal versus CHF using both eyes; and (9) Normal versus UHF using both eyes. Examining the measures of the left eye, right eye and both eyes separately investigate any asymmetries or distinctive features that each eye might bring to the categorization process. By looking at each eye separately, we can find characteristics unique to each eye that may be important for correctly classifying heart failure. Combining measurements from both eyes may also provide a more complete view, and by utilizing complimentary data from both sides, classification performance may be improved. This methodology guarantees a comprehensive assessment of the contribution of retinal OCT measurements from individual eyes to the identification and categorization of heart failure.

All experiments used a single GeForce NVIDIA MX350 (2 GB) graphics card to train and validate the models with an Intel Core i5-1135G7 (11th Gen) processor. The six machine learning methods we employed in each experiment were Decision Tree (DT) (36), Artificial Neural Network (ANN) (37), Random Forest (RF) (38), Extreme Gradient Boosting (XGBoost) (39), CatBoost (40), and Logistic Regression (LR) (41). The data is split into 80% for training and 20% for testing. The GridSearchCV utility in Python was utilized to modify hyperparameters through layered cross-validation, utilizing the Scikit-learn package (42). These hyperparameter values were selected based on a combination of domain expertise and experimentation, and Table 2 contains all of the tuned parameters for the ML models.

**Table 2:** Classification models and hyperparameters tuning.

Classification model	Hyperparameters
Decision tree	Max depth: [3, 5, 7, 10] Min. samples leaf: [10, 20, 50, 100] Criterion: [gini, entropy] Splitter: [best, random]
Artificial neural network	Hidden layer sizes: [50, 100] Activation: [logistic, relu] Solver: [lbfgs, adam] Alpha: [0.0001, 0.01, 0.1]
Random forest	Max depth: [10, 20, 30] Min samples leaf: [1, 14] Min samples split: [5, 12] Estimators: [100, 200] Random state: [42] Criterion: [gini, entropy]
Logistic regression	Max iteration: [100, 500, 1000] Solver: [liblinear, lbfgs] Penalty: [12, 16] C: [0.1, 1.0, 10.0]
CatBoost	Depth: [4, 16] Learning rate: [0.01, 0.1] Iterations: [100, 500] Subsample: [0.5, 0.8]
Extreme gradient boosting	Max depth: [4, 5] Estimators: [60] Learning rate: [0.01, 0.1] Subsample: [1.0] Colsample bytree: [0.5, 1.0] Lambda: [0.5, 1.0]

A Multi-Layer Perceptron (MLP) classifier trained on retinal OCT measurements is used in this study to evaluate feature significance and diagnose heart failure types. The absolute values of the coefficients associated with the input features are averaged to indicate their relative contributions to the model’s decision-making process, and the weights of the MLP model are analyzed to determine the significance of each feature. This method improves the machine learning model’s interpretability while highlighting the retinal parameters with the most predictive power for heart failure. Feature significance is a key component of explainable machine learning, as it sheds light on which retinal measurements are important for categorization. It enables physicians to comprehend the relationship between many features and the identification of heart failure, enabling improved patient management decision-making.

### 2.6 Performance evaluation metrics

We used several important performance indicators, such as Accuracy, Precision, Sensitivity, F1 Score, Matthews Correlation Coefficient (MCC), *p*-value, and Area Under the Receiver Operating Characteristic Curve (ROC AUC), to assess our classification models. Based on retinal OCT measurements, these metrics offer a thorough knowledge of how well the models identify heart failure. For every model, we used a 5-fold cross-validation technique to guarantee the reliability of our findings. This method divides the dataset into five subgroups, trains the model on four of them, and then validates it on the fifth, allowing for a more accurate estimation of model performance. This process is done five times to reduce the possibility of overfitting, with each subset acting as a validation set once.

## 3 Results

Supplementary Table S2 presents a comprehensive summary of the statistical analysis results for the demographic parameters and the retinal optical coherence tomography (OCT) measurements used in this study, covering data from both left and right eyes. To determine the statistical significance between the Normal group and each of the three heart failure types, *p*-values were computed for each parameter. Pairwise t-tests were conducted to evaluate group variations, with *p*-values indicating whether meaningful differences exist between the Normal group and each heart failure category.

**Figure 1:**
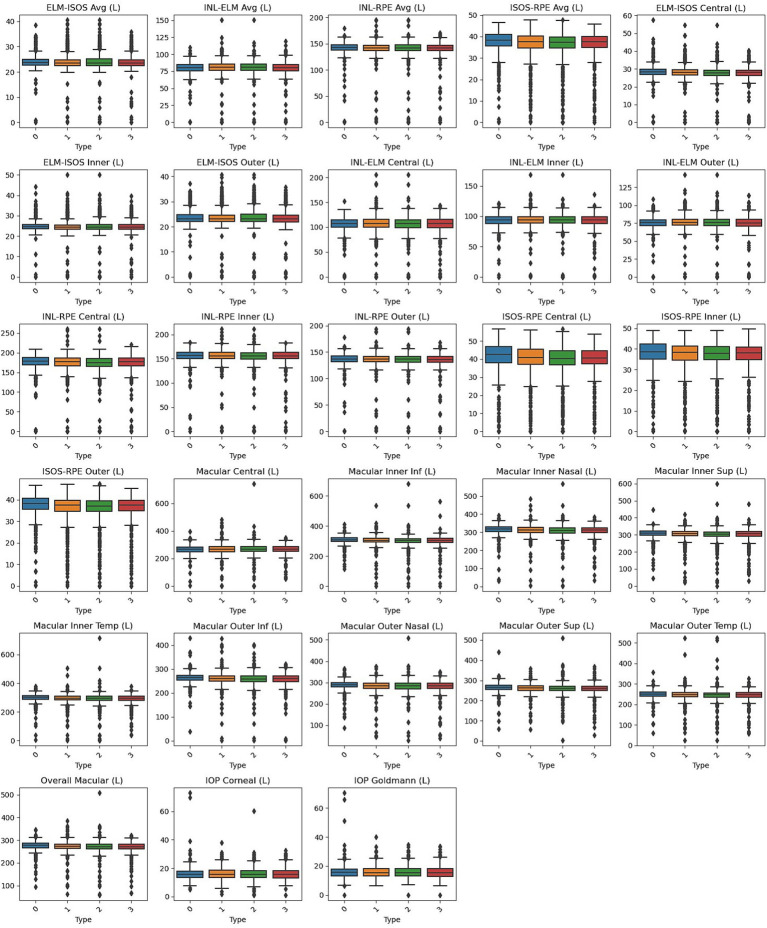
Box plots of left eye parameters by heart failure type. Normal (Type 0), Left Ventricle Heart Failure (Type 1), Congestive Heart Failure (Type 2), and Unspecified Heart Failure (Type 3).

**Figure 2:**
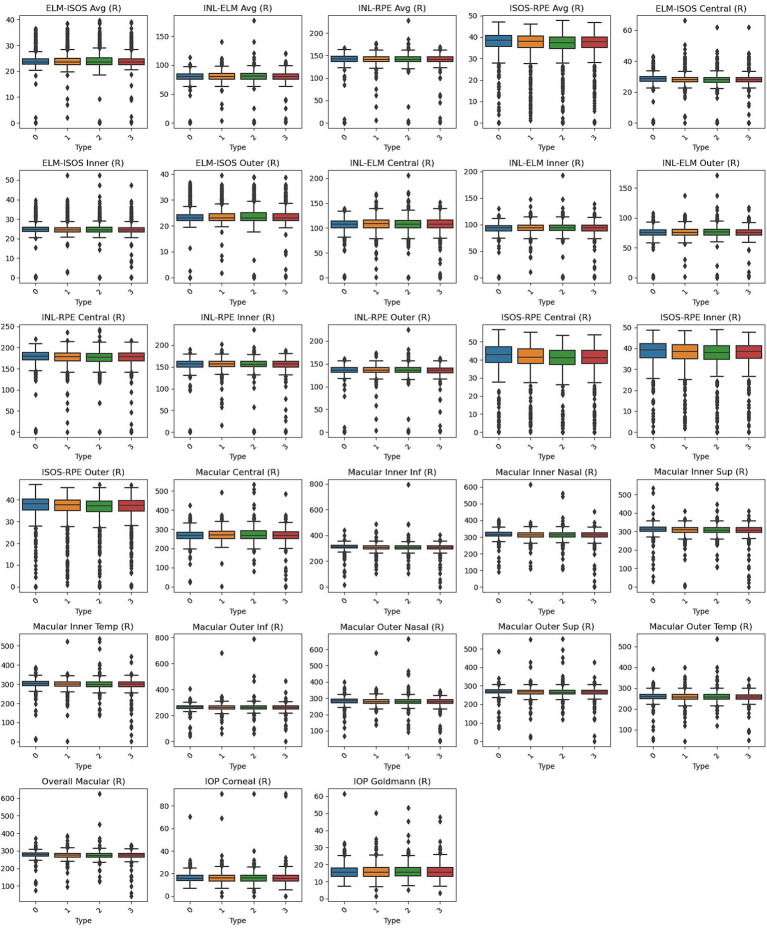
Box plots of right eye parameters by heart failure type. Normal (Type 0), Left Ventricle Heart Failure (Type 1), Congestive Heart Failure (Type 2), and Unspecified Heart Failure (Type 3).

Box plots, shown in Figures 1, 2, visually represent the distribution of retinal OCT measurements for the left and right eyes, respectively. These plots compare the Normal group with the three heart failure types, displaying the median, interquartile ranges, and any outliers. This visual summary highlights the spread and variability of retinal parameters across the groups, offering more profound insight into the observed differences.

All models were trained using these features. In the results section, the average performance metrics of the six models are presented in the tables below, with all models using the optimal combination of hyperparameters. In these tables, each column represents a distinct regression model. Table 3 shows the performance of detecting each type of heart failure from normal (control) individuals using only the left eye retinal OCT measurements. Table 4 displays the performance metrics for detecting HF types using only the right eye retinal OCT measurements. Lastly, Table 5 represents the performance of detecting HF types from normal individuals using both left and right eye retinal OCT measurements. These comprehensive results demonstrate the classification performance across different combinations of retinal OCT data, helping to evaluate the models’ abilities to differentiate between normal and heart failure conditions.

**Table 3:** Performance of different ML techniques using left retinal OCT features only.

Left retinal OCT features		DT	ANN	RF	LR	CatBoost	XGBoost
Normal vs. Congestive heart failure	Accuracy (%)	56.2	58.7	58.0	**63.0**	60.5	56.6
	Precision (%)	54.4	58.5	56.3	64.3	58.9	55.3
	Sensitivity (%)	63.5	52.6	62.0	54.0	62.8	56.9
	F1 Score (%)	58.6	55.4	59.0	58.7	60.8	56.1
	MCC	0.129	0.173	0.163	0.260	0.211	0.132
	*p*-value	0.0406	0.0055	0.0092	2.32E-05	0.0006	0.04
	ROC AUC	0.593	0.619	0.627	0.641	0.642	0.601
Normal vs. Left ventricle heart failure	Accuracy (%)	56.1	55.4	55.7	**56.4**	54.3	54.3
	Precision (%)	57.0	62.5	57.6	57.5	55.9	56.1
	Sensitivity (%)	60.4	33.6	53.7	59.1	54.4	52.3
	F1 Score (%)	58.6	43.7	55.6	58.3	55.1	54.2
	MCC	0.119	0.135	0.116	0.126	0.086	0.088
	*p*-value	0.057	0.030	0.065	0.042	0.176	0.169
	ROC AUC	0.557	0.552	0.612	0.612	0.602	0.575
Normal vs. Unspecified heart failure	Accuracy (%)	55.8	57.2	60.9	**65.6**	61.2	60.1
	Precision (%)	51.0	52.2	57.1	62.6	57.4	56.3
	Sensitivity (%)	78.6	75.4	57.1	61.1	58.7	57.1
	F1 Score (%)	61.9	61.7	57.1	61.8	58.0	56.7
	MCC	0.166	0.183	0.211	0.305	0.220	0.198
	*p*-value	0.0086	0.0036	0.0007	7.53E-07	0.0004	0.0015
	ROC AUC	0.593	0.641	0.666	0.677	0.668	0.639

Maximum accuracy is indicated in bold.

**Table 4:** Performance of different ML techniques using right retinal OCT features only.

Right retinal OCT features		DT	ANN	RF	LR	CatBoost	XGBoost
Normal vs. Congestive heart failure	Accuracy (%)	57.7	58.0	60.9	**66.2**	61.9	61.9
	Precision (%)	54.7	55.8	59.4	65.9	60.4	60.4
	Sensitivity (%)	75.9	66.4	62.0	63.5	63.5	63.5
	F1 Score (%)	63.6	60.7	60.7	64.7	61.9	61.9
	MCC	0.173	0.166	0.218	0.323	0.239	0.239
	p-value	0.006	0.0076	0.00042	1.19E-07	0.0001	0.0001
	ROC AUC	0.628	0.602	0.679	0.701	663	0.667
Normal vs. Left ventricle heart failure	Accuracy (%)	53.3	54.3	51.6	**58.1**	55.7	53.3
	Precision (%)	54.9	56.0	52.9	59.0	56.9	54.6
	Sensitivity (%)	52.3	53.0	54.4	61.7	58.4	55.7
	F1 Score (%)	53.6	54.5	53.6	60.3	57.6	55.1
	MCC	0.066	0.087	0.029	0.161	0.113	0.064
	*p*-value	0.313	0.172	0.703	0.009	0.072	0.330
	ROC AUC	0.545	0.579	0.559	0.612	0.582	0.563
Normal vs. Unspecified heart failure	Accuracy (%)	52.9	59.4	63.4	**64.5**	62.7	59.8
	Precision (%)	48.9	55.8	58.6	60.0	58.0	55.2
	Sensitivity (%)	71.4	53.2	67.5	66.7	65.9	62.7
	F1 Score (%)	58.1	54.5	62.7	63.2	61.7	58.7
	MCC	0.093	0.179	0.274	0.292	0.258	0.200
	*p*-value	0.1586	0.0043	9.44E-06	2.20E-06	3.13E-05	0.001
	ROC AUC	0.552	0.632	0.683	0.680	0.686	0.657

Maximum accuracy is indicated in bold.

**Table 5:** Performance of different ML techniques using both retinal OCT features only.

Left and right retinal OCT features		DT	ANN	RF	LR	CatBoost	XGBoost
Normal vs. Congestive heart failure	Accuracy (%)	55.5	60.1	61.9	61.9	60.1	**63.3**
	Precision (%)	54.6	59.5	61.7	59.4	58.7	61.6
	Sensitivity (%)	51.8	56.9	57.7	69.3	61.3	65.7
	F1 Score (%)	53.2	58.2	59.6	64.0	60.0	63.6
	MCC	0.109	0.202	0.237	0.244	0.203	0.268
	*p*-value	0.088	0.001	0.0001	7.03E-05	0.001	1.21E-05
	ROC AUC	0.605	0.609	0.667	0.681	0.657	0.664
Normal vs. Left ventricle heart failure	Accuracy (%)	52.6	52.2	**57.8**	54.7	54.3	54.7
	Precision (%)	54.1	53.3	58.4	56.2	55.8	56.2
	Sensitivity (%)	53.7	59.1	63.1	55.0	55.0	55.0
	F1 Score (%)	53.9	56.1	60.6	55.6	55.4	55.6
	MCC	0.051	0.041	0.153	0.093	0.086	0.093
	*p*-value	0.452	0.563	0.013	0.143	0.179	0.143
	ROC AUC	0.533	0.511	0.614	0.604	0.593	0.576
Normal vs. Unspecified heart failure	Accuracy (%)	57.2	57.6	60.1	**67.4**	61.6	59.1
	Precision (%)	52.8	52.5	56.3	64.5	57.2	54.7
	Sensitivity (%)	59.5	74.6	56.3	63.5	62.7	60.3
	F1 Score (%)	56.0	61.6	56.3	64.0	59.8	57.4
	MCC	0.148	0.187	0.197	0.342	0.233	0.182
	p-value	0.019	0.003	0.002	2.68E-08	0.000	0.004
	ROC AUC	0.582	0.648	0.667	0.692	0.686	0.643

Maximum accuracy is indicated in bold.

**Figure 3:**
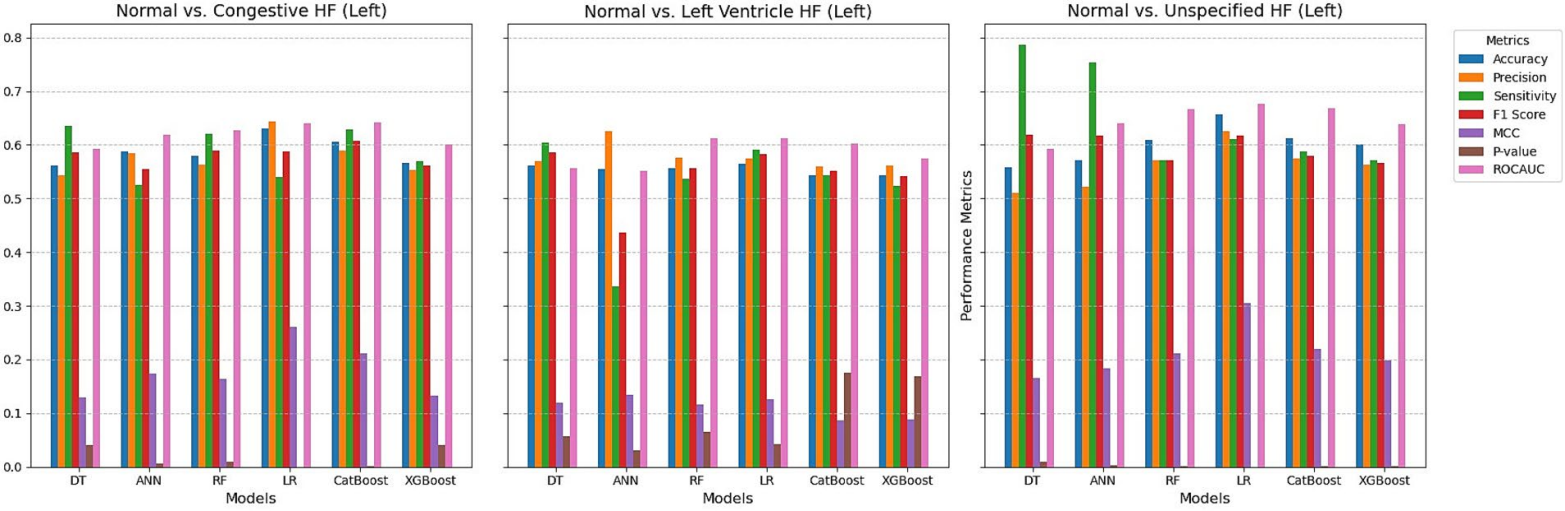
Comparing ML techniques using left retinal OCT features only.

The performance of the six models for detecting heart failure types using right retinal OCT measurements is shown in Figure 3. The models achieved accuracy ranging from 56.2 to 63.0% for distinguishing normal individuals from those with congestive heart failure. Logistic Regression outperformed the others, with an accuracy of 63.0%, precision of 64.3%, and ROC AUC of 0.641. The highest F1 score was 60.8% using CatBoost, while LR also achieved the highest MCC of 0.260. Except for the Tree model, all models demonstrated statistically significant differences with *p*-values below 0.05. In the case of detecting left ventricular heart failure, the models exhibited more modest performances, with accuracy values between 54.3 and 56.4%. Precision was highest for the ANN at 62.5%, while the best sensitivity was observed with the DR (Tree) model at 60.4%. F1 scores were generally lower than those for CHF detection, with LR performing at 58.3%. However, *p*-values indicated borderline significance for several models, and the ROC AUC values showed moderate discriminatory power, with the highest AUC of 0.612 for both LR and CatBoost. For distinguishing normal individuals from those with unspecified heart failure (UHF), the models showed higher accuracy overall, ranging from 55.8 to 65.6%, with LR again providing the best accuracy at 65.6% and MCC at 0.305. The highest ROC AUC value was 0.677 for LR, and p-values for all models indicated strong statistical significance, with the lowest being 7.53E-07 for LR. Regarding sensitivity, the DT model achieved the highest at 78.6%, although LR balanced precision and sensitivity effectively with a strong F1 score of 61.8%.

**Figure 4:**
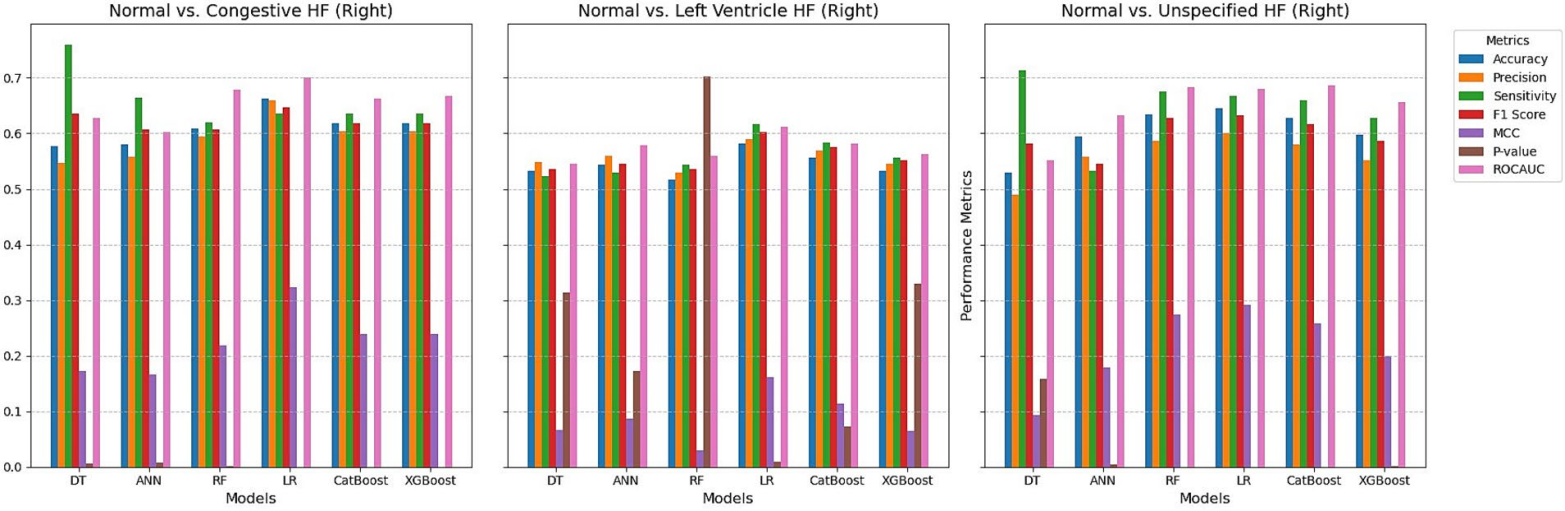
Comparing ML techniques using right retinal OCT features only.

Whereas, using right retinal OCT measurements for distinguishing normal individuals from those with congestive heart failure (Figure 4), the models achieved accuracy ranging from 57.7 to 66.2%, with Logistic Regression performing the others, having the highest accuracy of 66.2%, with precision of 65.9%, and ROC AUC of 0.701. The highest F1 score was also observed with LR at 64.7%, and LR achieved the highest MCC of 0.323. All models demonstrated statistically significant *p*-values, with LR having the lowest *p*-value at 1.19E-07. The models detecting left ventricular heart failure showed moderate performance, with accuracy values between 51.6 and 58.1%. LR again performed best, with an accuracy of 58.1%, an F1 score of 60.3%, and the highest MCC of 0.161. However, several models had *p*-values indicating weaker statistical significance, with only LR showing a significant *p*-value of 0.009. ROC AUC values were moderate, with LR achieving the highest AUC at 0.612. For distinguishing normal individuals from those with unspecified heart failure, accuracy ranged from 52.9 to 64.5%, with LR achieving the highest accuracy at 64.5% and the best F1 score of 63.2%. The highest ROC AUC value was 0.686 for CatBoost, and *p*-values indicated strong statistical significance for all models except Tree. LR had the highest MCC at 0.292, and sensitivity was also high for all models, with values above 62.7%.

**Figure 5:**
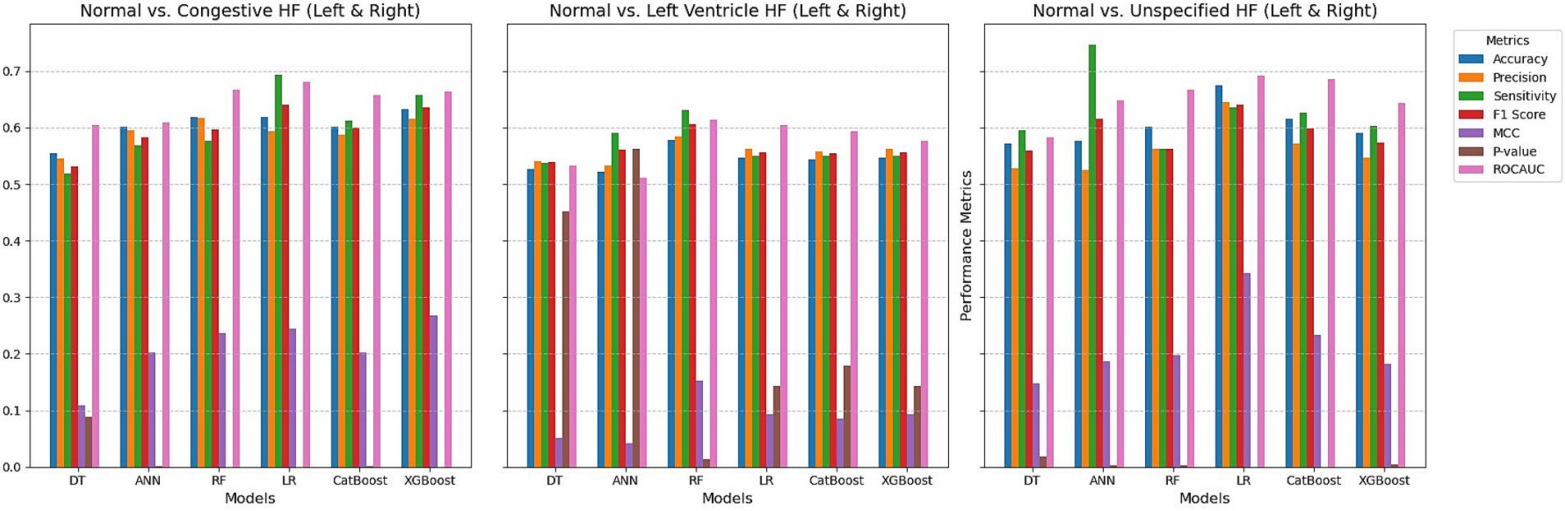
Comparing ML techniques using left and right retinal OCT features only.

Furthermore, using both left and right retinal OCT measurements to distinguish normal individuals from those with congestive heart failure (Figure 5), accuracy values ranged from 55.5 to 63.3%, with XGBoost achieving the highest accuracy of 63.3%. XGBoost also demonstrated the best performance in precision, at 61.6%, sensitivity at 65.7%, F1 score at 63.6%, and MCC at 0.268. Additionally, XGBoost had the most statistically significant *p*-value, 1.21E-05, and ROC AUC values were generally strong across the models, with Logistic Regression achieving the highest ROC AUC of 0.681. For detecting left ventricular heart failure, the accuracy values varied between 52.2 and 57.8%, with Random Forest performing the best, achieving an accuracy of 57.8%. RF also recorded the highest F1 score, 60.6%, and MCC, 0.153. However, *p*-values for this comparison indicated that only RF demonstrated statistical significance *p* = 0.013. ROC AUC scores ranged from 0.511 to 0.614, with RF again leading. The models performed particularly well in detecting unspecified heart failure, with LR achieving the highest accuracy of 67.4% and an F1 score of 64.0%. LR also had the highest MCC, 0.342, and the strongest p-value, 2.68E-08. The highest ROC AUC was also achieved by LR at 0.692. Across the board, models displayed strong performance in distinguishing normal individuals from those with UHF.

**Figure 6:**
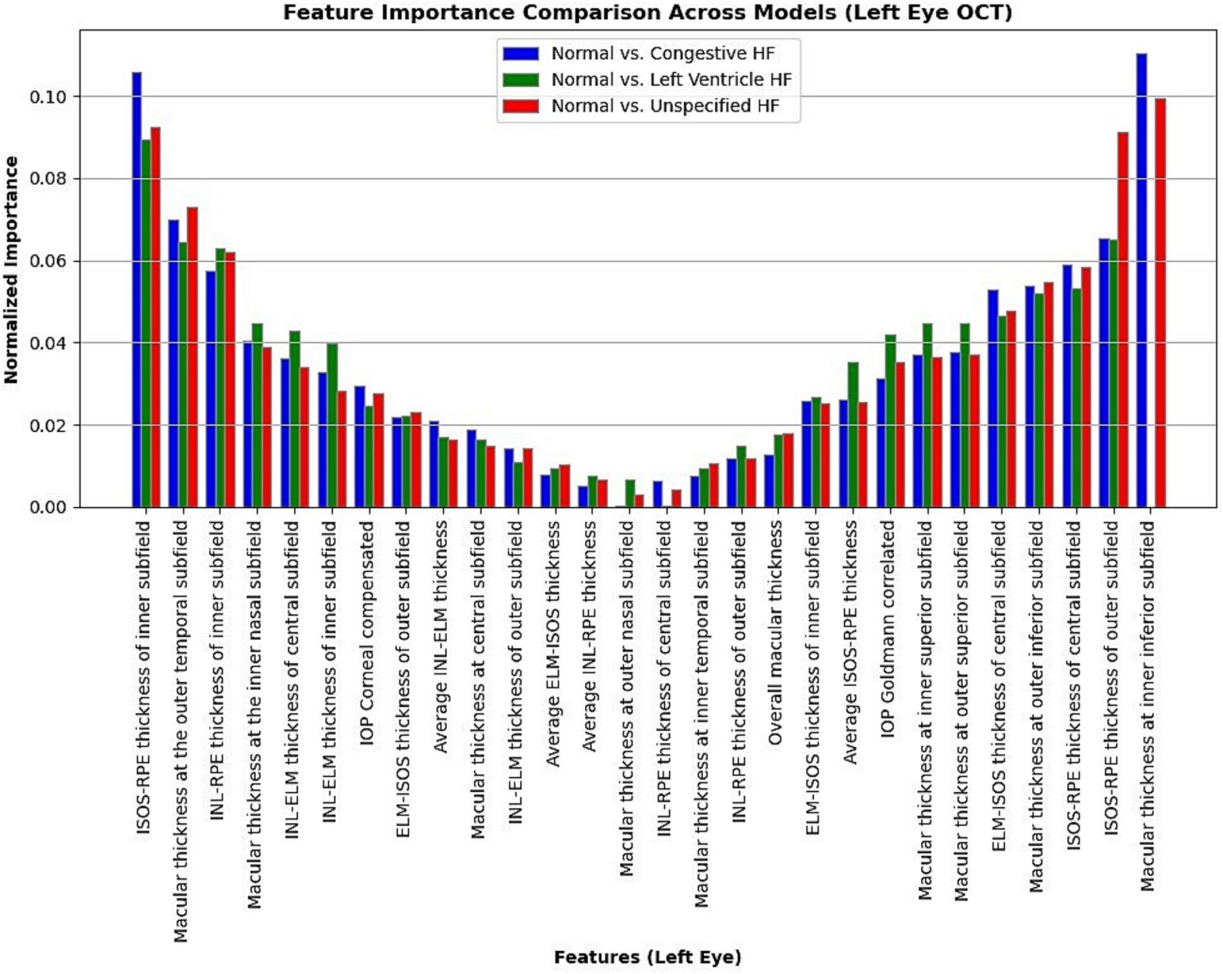
Normalized feature importance over the logistic regression models utilizing left retinal OCT features only.

**Figure 7:**
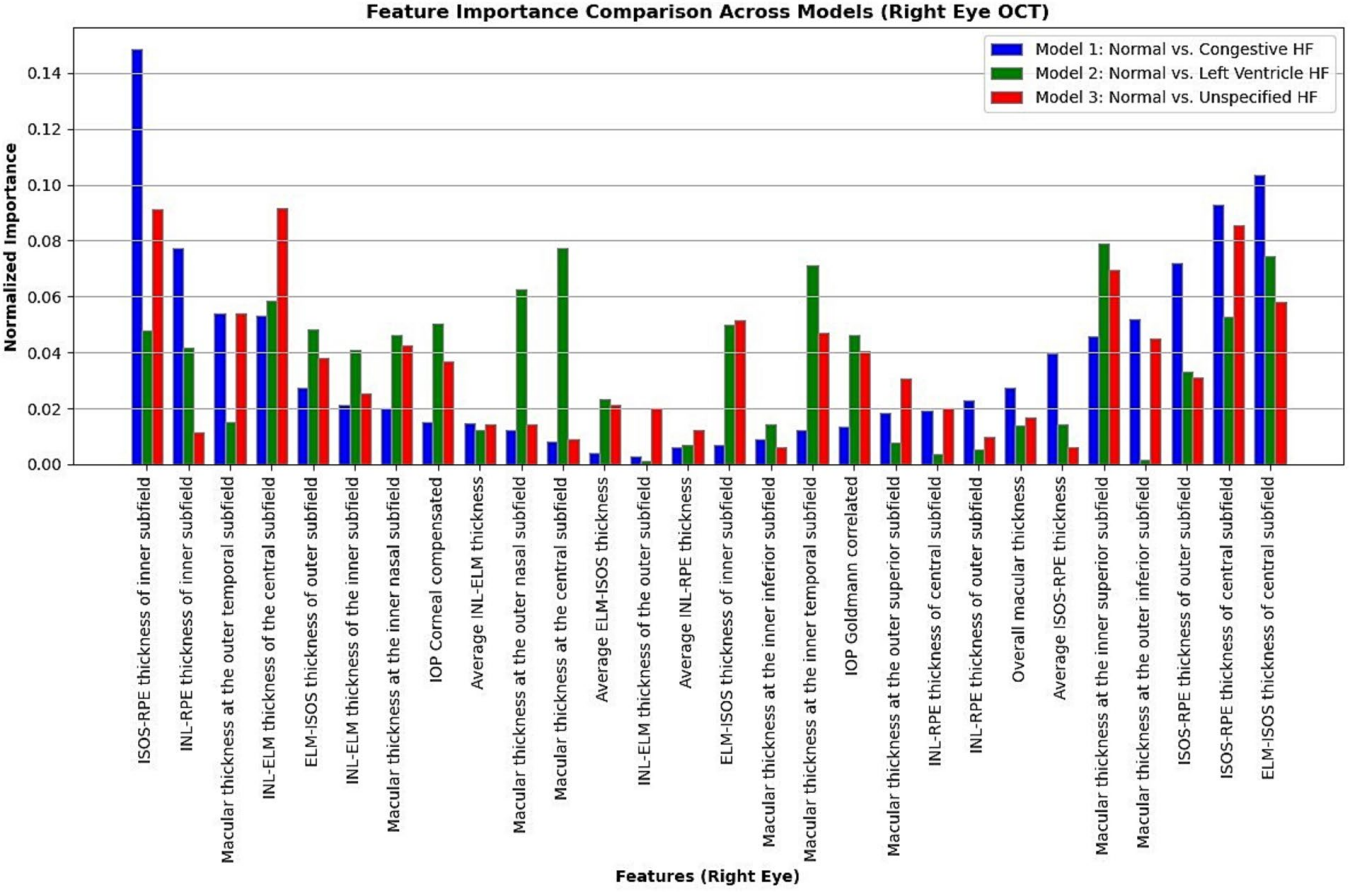
Normalized feature importance over the logistic regression models utilizing right retinal OCT features only.

**Figure 8:**
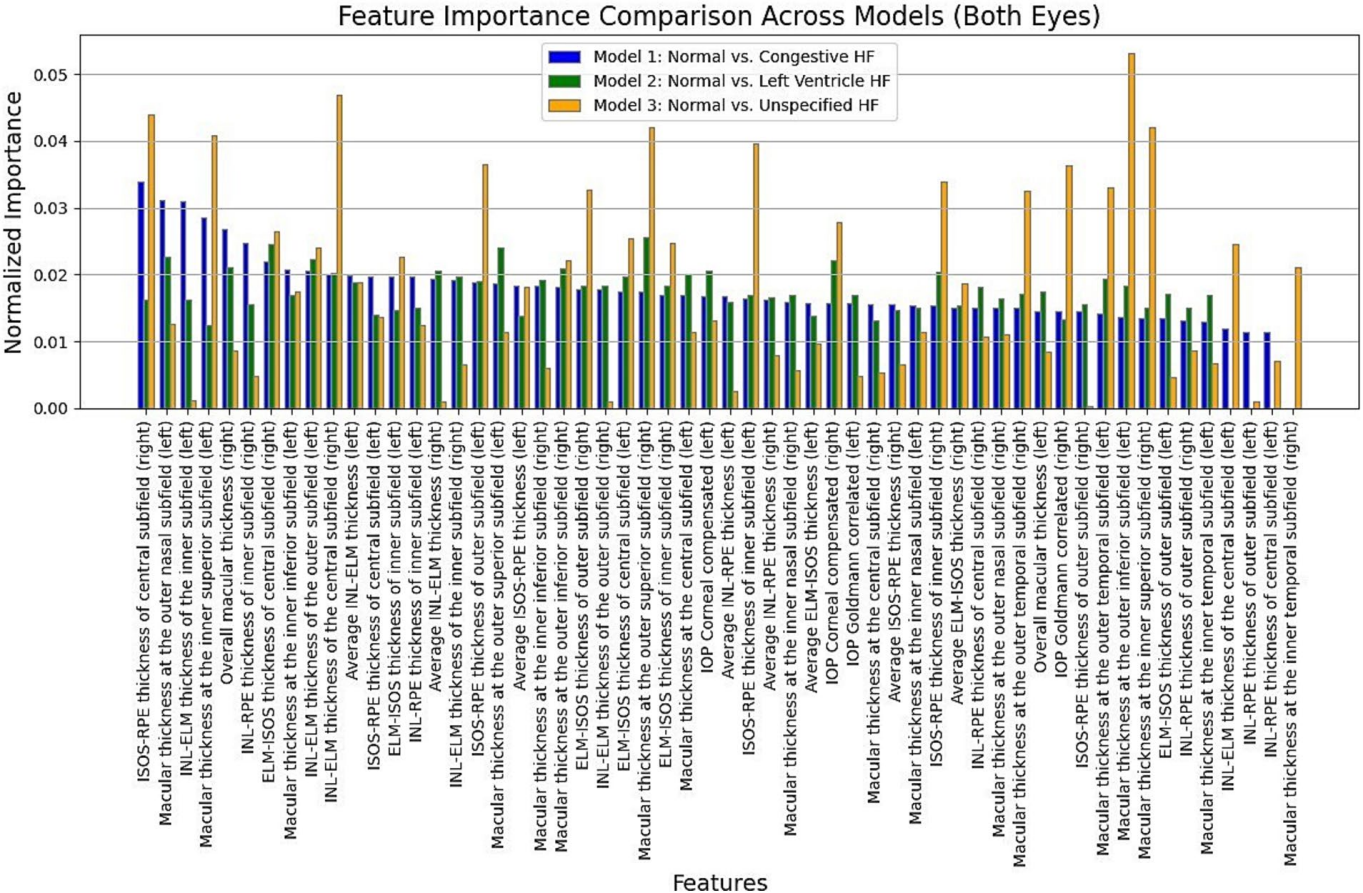
Normalized feature importance over the XGBoost, RF, and logistic regression models utilizing both retinal OCT features.

**Figure 9:**
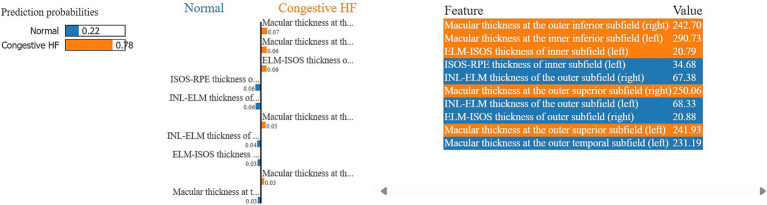
A visualization of LIME model scores (patient ID 2) over XGBoost model in classifying normal and congestive heart failure patients, utilizing both retinal OCT features.

**Figure 10:**
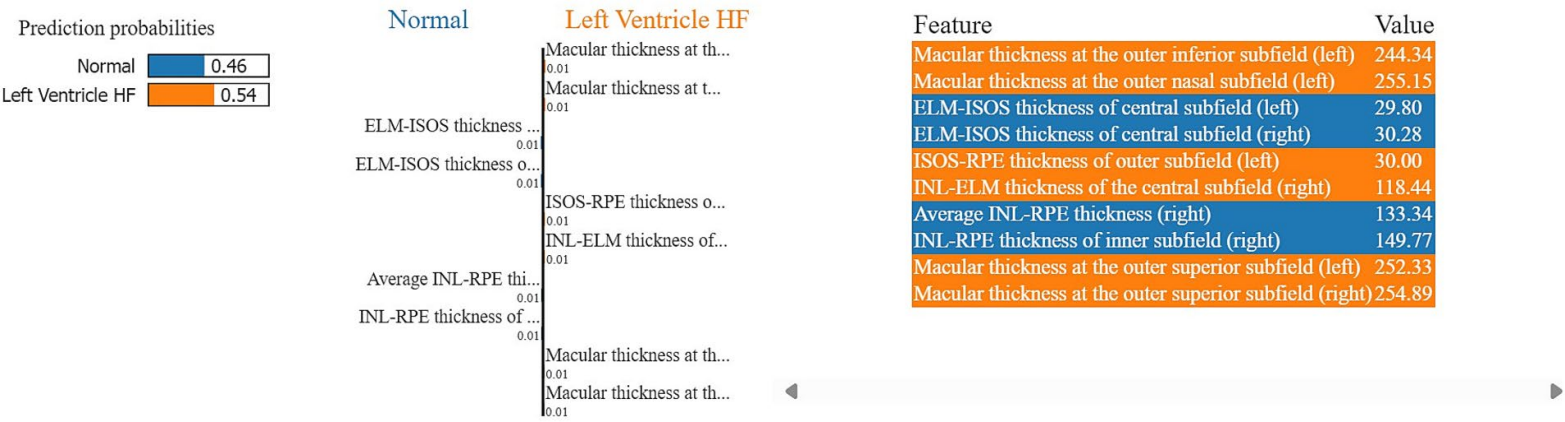
A visualization of LIME model scores (patient ID 5) over RF model in classifying normal and left ventricle heart failure patients, utilizing both retinal OCT features.

**Figure 11:**
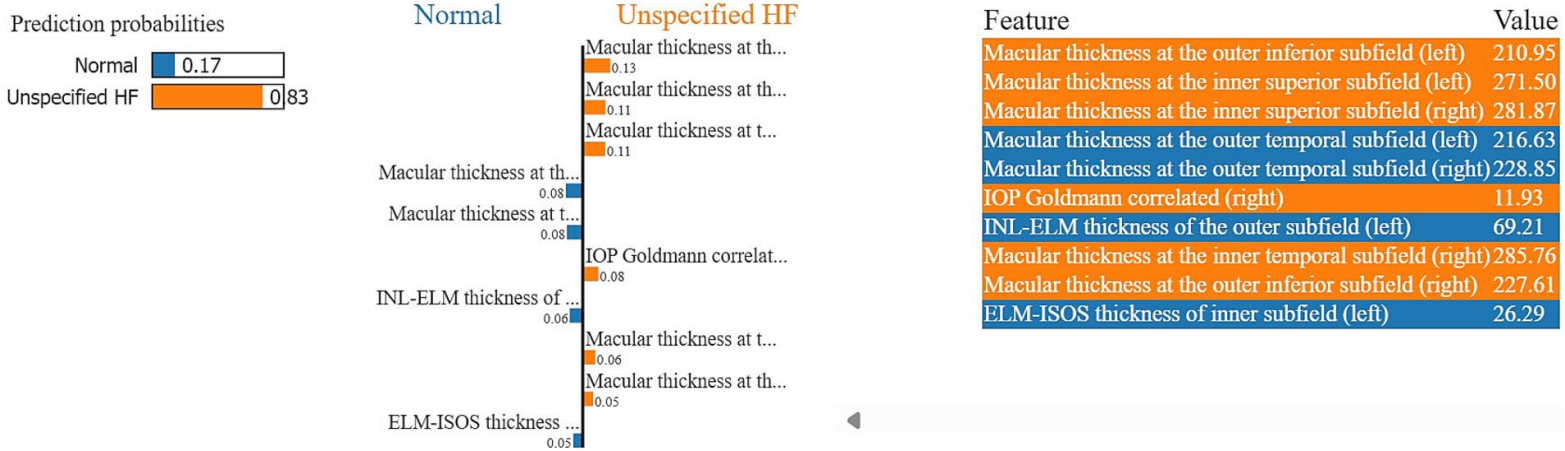
A visualization of LIME model scores (patient ID 7) over LR model in classifying normal and unspecified heart failure patients, utilizing both retinal OCT features.

Moreover, in this study, we employed the best classification models to evaluate the relevance of retinal OCT features from the left, right, and combined eyes. The results are illustrated in Figures 6–8, which highlight the significance of each feature in predicting heart failure type. Feature importance values were computed based on their impact on the target variable, providing valuable insights into the key factors driving the model’s predictions. In addition, the Local Interpretable Model-agnostic Explanations (LIME) algorithm was applied to enhance the local interpretability of models, utilizing OCT from both eyes for the prediction process in the XGBoost, RF, and LR models (proven to provide best results), as shown in Figures 9–11. Providing valuable insights into the model’s decision-making process for congestive, left ventricle, and unspecified HF, respectively. The use of explainable ML offers significant clinical implications. By identifying and ranking the most influential retinal features, clinicians can focus on key biomarkers more strongly associated with heart failure progression or detection. For instance, if a specific macular or retinal layer thickness proves to be a dominant factor, it could be integrated into diagnostic protocols, potentially improving early diagnosis and personalized treatment. This approach promotes trust in AI systems by allowing healthcare professionals to interpret and validate the model’s predictions.

## 4 Discussion

This study offers insightful information about retinal OCT features’ significance in detecting heart failure patients. The study also evaluates the significance of specific features in accurately distinguishing heart failure categories across the left, right, and both eyes through detailed analysis. Utilizing classification analysis, the research aims to enhance precision for optimal predictive performance. The investigation effectively attains elevated accuracy across all nine classification scenarios by identifying ideal hyperparameters and model configurations. These results showcase the capability of incorporating retinal OCT features into ML algorithms, indicating their potential as valuable tools for screening for heart failure diagnosis and treatment.

The retinal measures show significant differences between control subjects and those with various forms of heart failure. Remarkably, several retinal thickness metrics, including total macular and macular thickness in the superior and outer nasal subfields, consistently exhibit lower values in heart failure patients than controls, with *p*-values typically less than 0.005. Heart failure groups also have thinner central and inner subfield thicknesses, especially the ISOS-RPE layer. Parameters like ISOS-RPE and macular thickness at multiple subfields show significant reductions, reflecting possible retinal structural changes linked to heart failure. In contrast, other retinal measurements, such as ELM-ISOS and INL-ELM thicknesses, show non-significant differences between groups. According to these results, retinal measures may be useful biomarkers for differentiating between heart failure patients and healthy people. With the highest accuracy in differentiating between cases of normal from congestive (63.0%), left ventricle (56.4%), and unspecified heart failure (65.6%), logistic regression consistently outperformed other models when heart failure types were classified using left retinal OCT characteristics. The therapeutic promise of LR for identifying heart failure subgroups is suggested by its outstanding performance, which is demonstrated by significant *p*-values and remarkable precision. Additionally, CatBoost and XGBoost demonstrated strong performance, especially in congestive and unspecified cases, suggesting that retinal OCT features may be used as non-invasive indicators for the early detection and classification of heart failure, supporting individualized treatment plans.

Consistent performance across several models was found when right eye retinal OCT characteristics were analyzed for heart failure classification. The best results were obtained by Logistic Regression and CatBoost, especially when differentiating between healthy people and patients with congestive HF. LR had the highest accuracy (66.2%) and area under the curve (AUC) (0.701). Their high F1 scores, sensitivity, and accuracy further demonstrated these models’ resilience in identifying CHF. Although the models had difficulty achieving high accuracy in differentiating between patients with left ventricular heart failure and healthy individuals, LR and CatBoost outperformed the others, with LR exhibiting significant *p*-values of 0.009. The models performed poorly when distinguishing between patients with UHF; RF and XGBoost stood out with high accuracy.

Particularly for CHF and UHF, the combined left and right retinal OCT characteristics performed better in distinguishing between normal and heart failure types. XGBoost demonstrated its robustness by achieving the most fantastic accuracy (63.3%) and MCC (0.268) for CHF detection, along with a substantial *p*-value (1.21E-05) and ROC AUC (0.664). Additionally, LR demonstrated strong performance with an accuracy of 61.9% and an AUC of 0.681. With a noteworthy AUC (0.692) and the best accuracy (67.4%) and MCC (0.342) for UHF detection, LR demonstrated its efficacy in differentiating these individuals. All models performed poorly for LVHF; however, Random Forest had the best accuracy (57.8%) and MCC (0.153). Overall, the *p*-values were less significant, suggesting that it was more challenging to differentiate LVHF using OCT characteristics.

The feature importance analysis provides critical insights into distinguishing normal retinal characteristics from those associated with various heart failure conditions, including CHF, LVHF, and UHF. The most significant features in CHF classification include ISOS-RPE thickness in the inner subfield, INL-RPE thickness in the inner subfield, and macular thickness at the outer temporal subfield, that may indicate retinal stress, compromised blood flow, or fluid accumulation, which are commonly observed in CHF patients. Similarly, in LVHF classification, key features such as INL-ELM thickness in the central subfield, ELM-ISOS thickness in the outer subfield, and macular thickness at the inner nasal subfield may highlight ischemic damage, photoreceptor metabolic stress, or fluid retention, reflecting the impaired cardiac function associated with LVHF. For UHF classification, critical features include ISOS-RPE thickness in the central subfield, macular thickness at the outer nasal subfield, and INL-ELM thickness in the inner subfield, which are indicative of central retinal stress, peripheral retinal edema, or hypoxic damage, common in heart failure patients. These findings underscore the intricate relationship between retinal morphological changes and cardiovascular health, emphasizing the potential of retinal imaging as a non-invasive tool for heart failure detection and classification. Furthermore, explainable machine learning (ML) plays a crucial role in this analysis by providing transparency in model decision-making, ensuring clinicians can interpret and trust the predictions. By identifying the most influential retinal biomarkers for each heart failure type, explainable ML enhances the clinical applicability of AI-driven diagnostics, facilitating early detection, personalized risk assessment, and improved patient management.

Moreover, LIME results provide local interpretability, allowing ML models to provide explainable, patient-specific insights in complex medical applications, by highlighting the most influential features in each prediction. The model in Figure 9, predicts a 78% probability of congestive HF for patient ID 2. Primarily driven by macular thickness at the outer inferior (right) (242.7) and inner inferior (left) (290.7) subfields, along with ELM-ISOS of inner subfield thickness (left) (20.8). Whereas, ISOS-RPE thickness of inner subfield (left) (34.7) and INL-ELM thickness of the outer subfield (right) (67.4) strongly associated with the normal class. For the model in Figure 10, the patient ID 5 is 54% having left ventricle heart failure. Notably, macular at the outer inferior (244.3), and outer nasal (255.2) left subfields strongly contributing to the LVHF. Along with the ISOS-RPE outer (left) (30), and the INL-ELM central subfield (right) (118.44). Conversely, ELM-ISOS of the central subfields (left and right) (29.8, 30.3, respectively) were among the highest contributing factors to the normal class.

The model in Figure 11, predicts an 83% probability of unspecified heart failure for patient ID 7, primarily driven by macular thickness at various subfields, along with intraocular pressure goldmann correlated in the right eye (11.93). The outer inferior subfield macular thickness (left) and inner superior subfield macular thickness (left and right) were among the highest contributing factors. Conversely, features such as the outer temporal subfield macular thickness (left and right), INL-ELM (left) and ELM-ISOS (left) thickness had a stronger association with the normal class.

Propping further, to ensure the clinical translation of our findings, several key steps must be undertaken to validate and integrate retinal OCT biomarkers and ML models into routine medical practice:

1. Prospective Clinical Studies: A critical next step involves conducting large-scale, multi-center prospective studies to validate our ML models on diverse populations beyond the UK Biobank dataset. This would help assess model generalizability, reliability, and performance in real-world clinical settings.
2. Regulatory Compliance and Standardization: To facilitate clinical adoption, our approach must comply with regulatory standards (e.g., FDA, EMA, MHRA) for medical AI applications. This includes demonstrating model robustness, bias mitigation, and clinical benefit through randomized controlled trials.
3. Integration with Existing Diagnostic Pathways: The integration of retinal OCT-based ML models into cardiology and ophthalmology workflows requires seamless compatibility with electronic health record (EHR) systems and point-of-care diagnostics. This would allow for automated risk stratification of heart failure patients during routine ophthalmic exams.
4. Validation of Clinical Utility: Future research should focus on evaluating how retinal-based predictions improve patient outcomes, such as early detection rates, treatment optimization, and cost-effectiveness compared to conventional cardiac assessments.
5. Interdisciplinary Collaboration: Bridging cardiology, ophthalmology, and AI research will be essential for refining predictive algorithms, defining clinically relevant thresholds, and ensuring interpretability for physicians.

By following these steps, our study paves the way for retinal imaging as a scalable, non-invasive tool for heart failure screening and classification, ultimately enhancing early detection, personalized risk assessment, and precision medicine in cardiovascular care.

## 5 Limitations and future work

Although ML-based models effectively predict heart failure, our research recognizes many limitations. It is critical to recognize that no single parameter can accurately capture the whole spectrum of heart failure classification. For a more accurate and trustworthy evaluation, the intricacy of heart failure categorization necessitates a thorough evaluation of several parameters and diagnostic techniques. Our research highlights the necessity of a comprehensive strategy to guarantee reliable and clinically significant classification of individuals with heart failure. Moreover, although the present investigation employed a dataset encompassing patients from United Kingdom populations, subjecting the trained models to additional testing on more diverse patient groups is imperative to ensure broader applicability and generalization of their performance. In future investigations, the classification models formulated in this study might hold promise for adaptation or extension to predict diverse parameters associated with heart health, such as myocardial ischemia. However, their suitability for underlying conditions that may contribute to heart failure necessitates further research and validation.

Some demographic and clinical characteristics of heart failure groups (e.g., Age, Sex, BMI, HDL cholesterol, and Systolic Blood Pressure) found to be significantly different as compared to controls. Our study exclusively utilized retinal OCT measurements for predicting heart failure subtypes, intentionally excluding demographic and clinical characteristics such as age, and gender. This approach ensured that our models were solely driven by retinal parameters, such as ISOS-RPE and INL-ELM thickness, as non-invasive biomarkers for heart failure classification. While this strategy isolated the predictive power of retinal features, it inherently excluded confounding demographic variables, which could have provided additional context and improved model performance. The features identified by our explainable machine learning models as the most important for classification—such as reductions in ISOS-RPE and macular thickness—are strongly associated with microvascular dysfunction and structural changes linked to heart failure pathology. These alterations go beyond the gradual changes typically seen with aging, suggesting that the classification performance observed is predominantly driven by heart failure-related changes. Incorporating demographic and clinical characteristics, such as age and comorbidities, could further enhance model accuracy and robustness. Future work could integrate these variables alongside retinal OCT measurements to explore their combined predictive value.

To strengthen the clinical applicability of our findings, we propose avenues for future investigations such as focusing on longitudinal studies to track disease progression using retinal biomarkers. Conducting multi-center, long-term prospective studies will enable the continuous monitoring of retinal structural changes in correlation with heart failure progression, providing a deeper understanding of the temporal evolution of these biomarkers. This will help assess their predictive power in detecting early-stage cardiac dysfunction and evaluating the impact of therapeutic interventions over time.

Moreover, future studies could explore the integration of longitudinal retinal imaging data with machine learning models, leveraging sequential data analysis techniques such as recurrent neural networks (RNNs) and temporal convolutional networks (TCNs) to enhance predictive accuracy. Additionally, combining retinal biomarkers with other physiological indicators, such as ECG-derived circadian features, hemodynamic parameters, and serum biomarkers, may offer a more comprehensive and multi-modal approach for risk stratification and early detection of heart failure subtypes.

## 6 Conclusion

This study highlights the potential of using retinal OCT features as non-invasive biomarkers for detecting and classifying different types of heart failure. By analyzing and comparing retinal measurements from both the left and right eyes, the research successfully identifies key features that differentiate between normal individuals and heart failure patients, offering a novel approach to early diagnosis. The study achieves acceptable predictive accuracy through thorough classification analysis, showcasing the effectiveness of incorporating retinal features into machine learning algorithms. The study reveals significant differences in retinal thickness metrics between control subjects and heart failure patients, particularly in measures such as total macular thickness and ISOS-RPE thickness, which consistently showed reductions in heart failure patients. These findings suggest that structural changes in the retina may reflect the underlying pathophysiology of heart failure, further supporting the clinical relevance of these parameters as potential diagnostic tools.

The combined use of left and right eye features improved model performance, particularly for congestive and unspecified heart failure, where models like XGBoost and Logistic Regression achieved the highest accuracies. This highlights the importance of incorporating bilateral retinal data to enhance diagnostic precision. While the models demonstrated promising results for most heart failure subtypes, further research is needed to understand better the retinal changes associated with left ventricle heart failure, where the models performed less effectively. Moreover, this study highlights the critical role of explainable ML in distinguishing retinal biomarkers associated with different heart failure types, reinforcing the potential of retinal imaging as a non-invasive, cost-effective screening tool for early heart failure detection and classification. The feature importance analysis identifies key retinal parameters contributing to CHF, LVHF, and UHF classification, including ISOS-RPE thickness, INL-ELM thickness, macular thickness across various subfields, and intraocular pressure. Furthermore, LIME enhances local interpretability, ensuring model predictions remain transparent and clinically meaningful by identifying the most influential features in each classification. This patient-specific explanation strengthens trust in AI-driven diagnostics, supports early detection, personalized risk assessment, and improved patient management, bridging the gap between automated decision-making and clinical validation.

In conclusion, this study demonstrates the value of retinal OCT features in heart failure classification, emphasizing their potential as reliable, non-invasive indicators for early detection and personalized treatment strategies. By integrating these features into machine learning models, clinicians can improve heart failure diagnosis and management, offering a promising avenue for advancing cardiac care through ocular assessments.

## Data Availability

The data analyzed in this study is subject to the following licenses/restrictions: the research described in this study utilized the UK Biobank resource. However, we cannot publicly share the datasets generated and/or analyzed during this study due to their sensitive nature, potentially compromising research participants’ privacy. Nevertheless, interested parties may request access to the data from the corresponding author. Requests to access these datasets should be directed to Sona M. Al Younis, sonametep2@gmail.com.

## Footnotes

^1^ www.ukbiobank.ac.uk

## References

[1] Lindstrom M DeCleene N Dorsey H Fuster V Johnson CO LeGrand KE. Global burden of cardiovascular diseases and risks collaboration, 1990-2021. J Am Coll Cardiol. (2022) 80:2372–425. doi: 10.1016/j.jacc.2022.11.001, PMID: 36517116

[2] Alzaabi MA Abdelsalam A Alhammadi M Hani HB Almheiri A Al Matrooshi N. Evaluating biomarkers as tools for early detection and prognosis of heart failure: a comprehensive review. Card Fail Rev. (2024) 10:e06. doi: 10.15420/cfr.2023.24, PMID: 38915376 PMC11194781

[3] Al Younis SM Hadjileontiadis LJ Al Shehhi AM Stefanini C Alkhodari M Soulaidopoulos S. Investigating automated regression models for estimating left ventricular ejection fraction levels in heart failure patients using circadian ECG features. PLoS One. (2023) 18:e0295653. doi: 10.1371/journal.pone.0295653, PMID: 38079417 PMC10712857

[4] Al Younis SM Hadjileontiadis LJ Stefanini C Khandoker AH. Non-invasive technologies for heart failure, systolic and diastolic dysfunction modeling: a scoping review. Front Bioeng Biotechnol. (2023) 11:1261022. doi: 10.3389/fbioe.2023.1261022, PMID: 37920244 PMC10619666

[5] Tomasoni D Vitale C Guidetti F Benson L Braunschweig F Dahlström U. The role of multimorbidity in patients with heart failure across the left ventricular ejection fraction spectrum: data from the Swedish Heart failure registry. Eur J Heart Fail. (2024) 26:854–68. doi: 10.1002/ejhf.3112, PMID: 38131248

[6] Ben-Assuli O Heart T Klempfner R Padman R. Human-machine collaboration for feature selection and integration to improve congestive Heart failure risk prediction. Decis Support Syst. (2023) 172:113982. doi: 10.1016/j.dss.2023.113982

[7] Al Younis SM Hadjileontiadis LJ Khandoker AH Stefanini C Soulaidopoulos S Arsenos P. Prediction of heart failure patients with distinct left ventricular ejection fraction levels using circadian ECG features and machine learning. PLoS One. (2024) 19:e0302639. doi: 10.1371/journal.pone.0302639, PMID: 38739639 PMC11090346

[8] Nakao YM Nakao K Nadarajah R Banerjee A Fonarow GC Petrie MC. Prognosis, characteristics, and provision of care for patients with the unspecified heart failure electronic health record phenotype: a population-based linked cohort study of 95262 individuals. EClinicalMedicine. (2023) 63:102164. doi: 10.1016/j.eclinm.2023.102164, PMID: 37662516 PMC10474358

[9] Keane PA Grossi CM Foster PJ Yang Q Reisman CA Chan K. Optical coherence tomography in the UK biobank study–rapid automated analysis of retinal thickness for large population-based studies. PLoS One. (2016) 11:e0164095. doi: 10.1371/journal.pone.0164095, PMID: 27716837 PMC5055325

[10] Gutterman DD Chabowski DS Kadlec AO Durand MJ Freed JK Ait-Aissa K. The human microcirculation: regulation of flow and beyond. Circ Res. (2016) 118:157–72. doi: 10.1161/CIRCRESAHA.115.305364, PMID: 26837746 PMC4742348

[11] Barrot J Real J Vlacho B Romero-Aroca P Simó R Mauricio D. Diabetic retinopathy as a predictor of cardiovascular morbidity and mortality in subjects with type 2 diabetes. Front Med. (2022) 9:945245. doi: 10.3389/fmed.2022.945245, PMID: 36052329 PMC9424917

[12] Einarson TR Acs A Ludwig C Panton UH. Prevalence of cardiovascular disease in type 2 diabetes: a systematic literature review of scientific evidence from across the world in 2007–2017. Cardiovasc Diabetol. (2018) 17:1–19. doi: 10.1186/s12933-018-0728-6, PMID: 29884191 PMC5994068

[13] Liang K Gui S Wang X Wang Q Qiao J Tao L. Association of diabetic retinopathy on all-cause and cause-specific mortality in older adults with diabetes: National Health and nutrition examination survey, 2005–2008. Sci Rep. (2024) 14:10458. doi: 10.1038/s41598-024-58502-z, PMID: 38714673 PMC11076637

[14] Xu X-H Sun B Zhong S Wei D-D Hong Z Dong A-Q. Diabetic retinopathy predicts cardiovascular mortality in diabetes: a meta-analysis. BMC Cardiovasc Disord. (2020) 20:1–8. doi: 10.1186/s12872-020-01763-z, PMID: 33148188 PMC7643303

[15] Mir TA Arham AZ Fang W Alqahtani F Alkhouli M Gallo J. Acute vascular ischemic events in patients with central retinal artery occlusion in the United States: a nationwide study 2003-2014. Am J Ophthalmol. (2019) 200:179–86. doi: 10.1016/j.ajo.2019.01.009, PMID: 30689989 PMC6542256

[16] Kawasaki R Cheung N Wang JJ Klein R Klein BEK Cotch MF. Retinal vessel diameters and risk of hypertension: the multiethnic study of atherosclerosis. J Hypertens. (2009) 27:2386–93. doi: 10.1097/HJH.0b013e3283310f7e, PMID: 19680136 PMC2935621

[17] Smith W Wang JJ Wong TY Rochtchina E Klein R Leeder SR. Retinal arteriolar narrowing is associated with 5-year incident severe hypertension: the Blue Mountains eye study. Hypertension. (2004) 44:442–7. doi: 10.1161/01.HYP.0000140772.40322.ec, PMID: 15302843

[18] Tapp RJ Owen CG Barman SA Strachan DP Welikala RA Foster PJ. Retinal microvascular associations with cardiometabolic risk factors differ by diabetes status: results from the UK biobank. Diabetologia. (2022) 65:1652–63. doi: 10.1007/s00125-022-05745-y, PMID: 35852586 PMC9477904

[19] Hanssen H Streese L Vilser W. Retinal vessel diameters and function in cardiovascular risk and disease. Prog Retin Eye Res. (2022) 91:101095. doi: 10.1016/j.preteyeres.2022.101095, PMID: 35760749

[20] Streese L Khan AW Deiseroth A Hussain S Suades R Tiaden A. Physical activity may drive healthy microvascular ageing via downregulation of p66Shc. Eur J Prev Cardiol. (2020) 27:168–76. doi: 10.1177/2047487319880367, PMID: 31610708

[21] Gerrits N Elen B Van Craenendonck T Triantafyllidou D Petropoulos IN Malik RA. Age and sex affect deep learning prediction of cardiometabolic risk factors from retinal images. Sci Rep. (2020) 10:9432. doi: 10.1038/s41598-020-65794-4, PMID: 32523046 PMC7287116

[22] Al-Absi HRH Islam MT Refaee MA Chowdhury MEH Alam T. Cardiovascular disease diagnosis from DXA scan and retinal images using deep learning. Sensors. (2022) 22:4310. doi: 10.3390/s22124310, PMID: 35746092 PMC9228833

[23] Vaghefi E Squirrell D Yang S An S Xie L Durbin MK. Development and validation of a deep-learning model to predict 10-year atherosclerotic cardiovascular disease risk from retinal images using the UK biobank and EyePACS 10K datasets. Cardiovasc Digit Health J. (2024) 5:59–69. doi: 10.1016/j.cvdhj.2023.12.004, PMID: 38765618 PMC11096659

[24] Kadry S Dhanaraj RKKSK Manthiramoorthy C. Res-Unet based blood vessel segmentation and cardio vascular disease prediction using chronological chef-based optimization algorithm based deep residual network from retinal fundus images. Multimed Tools Appl. (2024):1–30. doi: 10.1007/s11042-024-18810-y

[25] Zhou Y Chia MA Wagner SK Ayhan MS Williamson DJ Struyven RR. A foundation model for generalizable disease detection from retinal images. Nature. (2023) 622:156–63. doi: 10.1038/s41586-023-06555-x, PMID: 37704728 PMC10550819

[26] Weerts J Raafs AG Sandhoefner B van der Heide FCT Mourmans SGJ Wolff N. Retinal vascular changes in Heart failure with preserved ejection fraction using optical coherence tomography angiography. J Clin Med. (2024) 13:1892. doi: 10.3390/jcm13071892, PMID: 38610657 PMC11012357

[27] Wang J. OCT image recognition of cardiovascular vulnerable plaque based on CNN. IEEE Access. (2020) 8:140767–76. doi: 10.1109/ACCESS.2020.3007599

[28] Chandra A Seidelmann SB Claggett BL Klein BE Klein R Shah AM. The association of retinal vessel calibres with heart failure and long-term alterations in cardiac structure and function: the atherosclerosis risk in communities (ARIC) study. Eur J Heart Fail. (2019) 21:1207–15. doi: 10.1002/ejhf.1564, PMID: 31373139 PMC12611558

[29] Poplin R Varadarajan AV Blumer K Liu Y McConnell MV Corrado GS. Prediction of cardiovascular risk factors from retinal fundus photographs via deep learning. Nat Biomed Eng. (2018) 2:158–64. doi: 10.1038/s41551-018-0195-0, PMID: 31015713

[30] Chaikijurajai T Ehlers JP Tang WHW. Retinal microvasculature: a potential window into heart failure prevention. Heart Fail. (2022) 10:785–91. doi: 10.1016/j.jchf.2022.07.004 36328644

[31] Khalilipur E Mahdizad Z Molazadeh N Faghihi H Naderi N Mehrabi Bahar M. Microvascular and structural analysis of the retina and choroid in heart failure patients with reduced ejection fraction. Sci Rep. (2023) 13:5467. doi: 10.1038/s41598-023-32751-w, PMID: 37015968 PMC10073248

[32] Naegele MP Barthelmes J Ludovici V Cantatore S von Eckardstein A Enseleit F. Retinal microvascular dysfunction in heart failure. Eur Heart J. (2018) 39:47–56. doi: 10.1093/eurheartj/ehx565, PMID: 29069316

[33] Collins R. (2007). UK biobank: protocol for a large-scale prospective epidemiological resource.

[34] Sudlow C Gallacher J Allen N Beral V Burton P Danesh J. UK biobank: an open access resource for identifying the causes of a wide range of complex diseases of middle and old age. PLoS Med. (2015) 12:e1001779. doi: 10.1371/journal.pmed.1001779, PMID: 25826379 PMC4380465

[35] Ko F Foster PJ Strouthidis NG Shweikh Y Yang Q Reisman CA. Associations with retinal pigment epithelium thickness measures in a large cohort: results from the UK biobank. Ophthalmology. (2017) 124:105–17. doi: 10.1016/j.ophtha.2016.07.033, PMID: 27720551

[36] Maimon OZ Rokach L. Data mining with decision trees: theory and applications World scientific (2014).

[37] Diaz GI Fokoue-Nkoutche A Nannicini G Samulowitz H. An effective algorithm for hyperparameter optimization of neural networks. IBM J Res Dev. (2017) 61:9:1–9:11. doi: 10.1147/JRD.2017.2709578, PMID: 33813791

[38] Yang L Wu H Jin X Zheng P Hu S Xu X. Study of cardiovascular disease prediction model based on random forest in eastern China. Sci Rep. (2020) 10:5245. doi: 10.1038/s41598-020-62133-5, PMID: 32251324 PMC7090086

[39] Wang Y Miao X Xiao G Huang C Sun J Wang Y. Clinical prediction of heart failure in hemodialysis patients: based on the extreme gradient boosting method. Front Genet. (2022) 13:889378. doi: 10.3389/fgene.2022.889378, PMID: 35559036 PMC9086166

[40] Wei X Rao C Xiao X Chen L Goh M. Risk assessment of cardiovascular disease based on SOLSSA-CatBoost model. Expert Syst Appl. (2023) 219:119648. doi: 10.1016/j.eswa.2023.119648

[41] Ambrish G Ganesh B Ganesh A Srinivas C Mensinkal K. Logistic regression technique for prediction of cardiovascular disease. Global Trans Proc. (2022) 3:127–30. doi: 10.1016/j.gltp.2022.04.008

[42] Cawley GC Talbot NLC. On over-fitting in model selection and subsequent selection bias in performance evaluation. J Machine Learn Res. (2010) 11:2079–107.

