# Supplementary material for: Prediction of heart failure risk factors from retinal optical imaging via explainable machine learning
Source: Front Med (Lausanne). 2025 Mar 17;12:1551557. doi: 10.3389/fmed.2025.1551557 (PMC11955505; doi:10.3389/fmed.2025.1551557)
Supplement: Supplementary file 1 [file Data_Sheet_1.docx]

Supplementary Material

# Supplement Table S1: Derived retinal optical coherence tomography features and their definitions

| **Category** | **Measurement** | **Physical Meaning** |
| --- | --- | --- |
| ELM-ISOS Thickness (Left/Right) | ELM-ISOS Average Thickness | Measures the thickness between the External Limiting Membrane (ELM) and Inner Segment-Outer Segment (ISOS) junction, critical for photoreceptor function. |
|  | ELM-ISOS Central Subfield | Represents the thickness of the ELM-ISOS junction specifically in the central subfield of the macula, related to central vision. |
|  | ELM-ISOS Inner Subfield | Measures the thickness of the ELM-ISOS junction in the inner subfield of the retina, surrounding the central subfield. |
|  | ELM-ISOS Outer Subfield | Captures the thickness of the ELM-ISOS junction in the outer subfield, further away from the central vision area. |
| INL-ELM Thickness (Left/Right) | INL-ELM Average Thickness | The thickness between the Inner Nuclear Layer (INL) and External Limiting Membrane (ELM), indicating the integrity of the retina's inner layers. |
|  | INL-ELM Central Subfield | Thickness between the INL and ELM in the central subfield, key for evaluating retinal layer function in central vision. |
|  | INL-ELM Inner Subfield | INL-ELM thickness in the inner region surrounding the central subfield. |
|  | INL-ELM Outer Subfield | INL-ELM thickness in the outer subfield of the macula. |
| INL-RPE Thickness (Left/Right) | INL-RPE Average Thickness | Measures the thickness between the Inner Nuclear Layer (INL) and Retinal Pigment Epithelium (RPE), important for evaluating retinal integrity. |
|  | INL-RPE Central Subfield | The INL-RPE thickness in the central macular region, linked to core visual function. |
|  | INL-RPE Inner Subfield | Thickness of the INL-RPE junction in the inner macular subfield. |
|  | INL-RPE Outer Subfield | INL-RPE thickness in the outer subfield, extending away from the fovea. |
| ISOS-RPE Thickness (Left/Right) | ISOS-RPE Average Thickness | Thickness between the Inner Segment-Outer Segment (ISOS) junction and the Retinal Pigment Epithelium (RPE), critical for assessing photoreceptor health. |
|  | ISOS-RPE Central Subfield | ISOS-RPE thickness in the central subfield of the retina is linked to visual acuity and central photoreceptor function. |
|  | ISOS-RPE Inner Subfield | Thickness of the ISOS-RPE junction in the inner subfield of the retina. |
|  | ISOS-RPE Outer Subfield | ISOS-RPE thickness in the outer subfield, representing peripheral photoreceptor function. |
| Macular Thickness | Macular Thickness at Central Subfield (Left/Right) | Central macular thickness is a key measurement in assessing macular health, particularly for central vision. |
|  | Macular Thickness at Inner Subfields (Inferior, Nasal, Superior, Temporal - Left/Right) | Thickness in various inner subfields of the macula is related to the health and function of areas surrounding the central macula. |
|  | Macular Thickness at Outer Subfields (Inferior, Nasal, Superior, Temporal - Left/Right) | Thickness in the outer macular subfields, extending further from the central vision area, is important for peripheral retinal health. |
|  | Overall Macular Thickness (Left/Right) | The average thickness across the entire macula gives an overall measure of macular health. |
| Intraocular Pressure (IOP) (Left/Right) | IOP Corneal Compensated | Measures intraocular pressure, adjusted for corneal thickness, important for assessing glaucoma risk. |
|  | IOP Goldmann Correlated | The Goldmann method measures intraocular pressure, the gold standard for glaucoma diagnosis. |

# Supplement Table S2: The demographic and clinical characteristics of Heart Failure groups.

| Parameter | Control | Type 1 | Type 2 | Type 3 | p-value ( Control vs. ) | | |
| --- | --- | --- | --- | --- | --- | --- | --- |
|  |  |  |  |  | Type 1 | Type 2 | Type 3 |
| Age(y) | 56 ± 8 | 62 ± 6 | 63 ± 5.5 | 62 ± 5.6 | **<0.005** | **<0.005** | **<0.005** |
| Sex (F/M) | 223/478 | 241/503 | 414/287 | 246/432 | **<0.005** | **<0.005** | **<0.005** |
| Body mass index (BMI) | 26.95 ± 4.93 | 29.54 ± 5.05 | 30.48 ± 5.84 | 29.67 ± 5.45 | **<0.005** | **<0.005** | **<0.005** |
| HDL cholesterol | 1.52 ± 0.39 | 1.31 ± 0.37 | 1.33 ± 0.39 | 1.35 ± 0.38 | **<0.005** | **<0.005** | **<0.005** |
| Systolic Blood Pressure | 138.17 ± 19.46 | 144.51 ± 19.98 | 146.41 ± 21.41 | 145.22 ± 20.79 | **<0.005** | **<0.005** | **<0.005** |
| Diastolic Blood Pressure | 81.44 ± 10.53 | 81.58 ± 11.62 | 82.07 ± 11.82 | 81.40 ± 11.34 | 0.799 | 0.291 | 0.948 |
| Left Eye Measurements | | | | | | | |
| Average ELM-ISOS thickness | 24.10 ± 3.01 | 24.10 ± 3.59 | 24.14 ± 3.93 | 23.82 ± 3.52 | 0.981 | 0.863 | 0.108 |
| Average INL-ELM thickness | 80.00 ± 8.68 | 80.74 ± 10.59 | 80.22 ± 11.62 | 79.84 ± 10.81 | 0.147 | 0.685 | 0.765 |
| Average INL-RPE thickness | 141.63 ± 12.88 | 141.10 ± 15.31 | 140.21 ± 17.85 | 140.04 ± 16.75 | 0.478 | 0.087 | **0.047** |
| Average ISOS-RPE thickness | 37.52 ± 5.59 | 36.26 ± 6.93 | 35.85 ± 7.12 | 36.37 ± 6.56 | **<0.005** | **<0.005** | **<0.005** |
| ELM-ISOS thickness of central subfield | 28.25 ± 3.93 | 27.88 ± 4.31 | 27.58 ± 4.53 | 27.41 ± 4.30 | 0.083 | **<0.005** | **<0.005** |
| ELM-ISOS thickness of inner subfield | 24.87 ± 3.09 | 24.74 ± 3.72 | 24.75 ± 4.10 | 24.44 ± 3.55 | 0.479 | 0.527 | **0.017** |
| ELM-ISOS thickness of outer subfield | 23.72 ± 3.13 | 23.77 ± 3.66 | 23.83 ± 3.99 | 23.50 ± 3.61 | 0.796 | 0.585 | 0.226 |
| INL-ELM thickness of the central subfield | 105.96 ± 14.77 | 106.18 ± 17.16 | 105.38 ± 18.10 | 105.25 ± 17.20 | 0.790 | 0.515 | 0.411 |
| INL-ELM thickness of the inner subfield | 92.78 ± 10.56 | 93.15 ± 12.67 | 92.70 ± 13.77 | 92.40 ± 12.85 | 0.551 | 0.902 | 0.545 |
| INL-ELM thickness of the outer subfield | 75.25 ± 8.40 | 76.12 ± 10.34 | 75.59 ± 11.33 | 75.18 ± 10.47 | 0.080 | 0.522 | 0.891 |
| INL-RPE thickness of central subfield | 175.50 ± 21.09 | 173.86 ± 22.99 | 172.04 ± 25.78 | 172.44 ± 25.23 | 0.159 | **<0.005** | **0.014** |
| INL-RPE thickness of inner subfield | 155.60 ± 15.19 | 154.90 ± 17.51 | 153.98 ± 20.33 | 153.87 ± 19.23 | 0.415 | 0.091 | 0.064 |
| INL-RPE thickness of outer subfield | 136.23 ± 12.49 | 135.80 ± 14.91 | 134.95 ± 17.31 | 134.74 ± 16.14 | 0.548 | 0.111 | 0.054 |
| ISOS-RPE thickness of central subfield | 41.29 ± 8.71 | 39.80 ± 9.39 | 39.08 ± 9.49 | 39.78 ± 8.69 | **<0.005** | **<0.005** | **<0.005** |
| ISOS-RPE thickness of inner subfield | 37.95 ± 6.55 | 37.00 ± 7.46 | 36.53 ± 7.84 | 37.03 ± 7.07 | **0.01** | **<0.005** | **0.012** |
| ISOS-RPE thickness of outer subfield | 37.26 ± 5.43 | 35.91 ± 6.89 | 35.53 ± 7.04 | 36.05 ± 6.53 | **<0.005** | **<0.005** | **<0.005** |
| Macular thickness at the central subfield | 266.01 ± 31.35 | 267.71 ± 36.26 | 267.34 ± 40.95 | 266.75 ± 32.99 | 0.343 | 0.495 | 0.671 |
| Macular thickness at the inner inferior subfield | 307.32 ± 24.67 | 302.28 ± 32.07 | 299.79 ± 37.65 | 300.81 ± 33.60 | **<0.005** | **<0.005** | **<0.005** |
| Macular thickness at the inner nasal subfield | 313.90 ± 28.60 | 310.74 ± 30.31 | 307.79 ± 33.85 | 308.09 ± 31.28 | **0.04** | **<0.005** | **<0.005** |
| Macular thickness at the inner superior subfield | 307.52 ± 29.66 | 302.69 ± 34.01 | 299.97 ± 38.60 | 299.87 ± 37.47 | **<0.005** | **<0.005** | **<0.005** |
| Macular thickness at the inner temporal subfield | 297.07 ± 29.55 | 293.20 ± 33.86 | 290.42 ± 40.91 | 290.54 ± 33.00 | **0.02** | **<0.005** | **<0.005** |
| Macular thickness at the outer inferior subfield | 263.50 ± 22.48 | 259.51 ± 27.84 | 258.12 ± 28.86 | 257.65 ± 27.09 | **<0.005** | **<0.005** | **<0.005** |
| Macular thickness at the outer nasal subfield | 287.37 ± 22.36 | 283.28 ± 25.86 | 282.20 ± 27.95 | 281.84 ± 28.46 | **<0.005** | **<0.005** | **<0.005** |
| Macular thickness at the outer superior subfield | 264.28 ± 22.77 | 260.52 ± 23.80 | 258.42 ± 27.68 | 258.23 ± 27.02 | **<0.005** | **<0.005** | **<0.005** |
| Macular thickness at the outer temporal subfield | 248.88 ± 21.45 | 246.15 ± 28.32 | 244.38 ± 32.20 | 244.64 ± 25.18 | **0.04** | **<0.005** | **<0.005** |
| Overall macular thickness | 275.00 ± 19.81 | 271.36 ± 23.58 | 269.56 ± 26.86 | 269.48 ± 23.81 | **<0.005** | **<0.005** | **<0.005** |
| IOP Corneal compensated | 15.95 ± 4.64 | 16.02 ± 4.04 | 16.02 ± 4.32 | 15.98 ± 4.01 | 0.755 | 0.789 | 0.917 |
| IOP Goldmann correlated | 15.90 ± 4.77 | 15.76 ± 4.00 | 15.79 ± 3.95 | 15.73 ± 4.06 | 0.557 | 0.659 | 0.484 |
| Right Eye Measurements | | | | | | | |
| Average ELM-ISOS thickness | 24.04 ± 3.33 | 24.23 ± 3.10 | 24.25 ± 3.62 | 24.08 ± 3.45 | 0.273 | 0.259 | 0.814 |
| Average INL-ELM thickness | 79.78 ± 9.60 | 80.63 ± 8.44 | 80.70 ± 11.01 | 80.12 ± 9.84 | 0.076 | 0.096 | 0.517 |
| Average INL-RPE thickness | 141.13 ± 14.44 | 141.45 ± 11.06 | 140.99 ± 15.39 | 140.69 ± 14.46 | 0.636 | 0.861 | 0.567 |
| Average ISOS-RPE thickness | 37.31 ± 6.26 | 36.60 ± 6.82 | 36.04 ± 6.92 | 36.48 ± 6.44 | **0.04** | **<0.005** | **0.015** |
| ELM-ISOS thickness of central subfield | 28.39 ± 3.75 | 28.11 ± 3.89 | 27.92 ± 4.29 | 27.94 ± 4.22 | 0.168 | **0.028** | **0.036** |
| ELM-ISOS thickness of inner subfield | 24.84 ± 3.31 | 24.92 ± 3.21 | 24.89 ± 3.71 | 24.68 ± 3.54 | 0.626 | 0.792 | 0.375 |
| ELM-ISOS thickness of outer subfield | 23.64 ± 3.44 | 23.88 ± 3.21 | 23.93 ± 3.73 | 23.77 ± 3.54 | 0.184 | 0.141 | 0.516 |
| INL-ELM thickness of the central subfield | 106.36 ± 14.45 | 107.29 ± 14.51 | 106.68 ± 16.74 | 106.95 ± 16.67 | 0.221 | 0.697 | 0.484 |
| INL-ELM thickness of the inner subfield | 92.60 ± 11.17 | 93.57 ± 9.89 | 93.43 ± 12.49 | 92.98 ± 12.22 | 0.081 | 0.193 | 0.548 |
| INL-ELM thickness of the outer subfield | 75.00 ± 9.38 | 75.80 ± 8.40 | 75.97 ± 10.90 | 75.32 ± 9.44 | 0.086 | 0.074 | 0.530 |
| INL-RPE thickness of central subfield | 176.64 ± 21.01 | 175.96 ± 19.15 | 174.69 ± 22.40 | 175.15 ± 23.17 | 0.518 | 0.093 | 0.212 |
| INL-RPE thickness of inner subfield | 155.36 ± 16.53 | 155.79 ± 12.97 | 155.24 ± 17.18 | 154.81 ± 17.48 | 0.575 | 0.893 | 0.551 |
| INL-RPE thickness of outer subfield | 135.60 ± 13.95 | 135.92 ± 10.80 | 135.53 ± 15.07 | 135.23 ± 13.75 | 0.623 | 0.920 | 0.613 |
| ISOS-RPE thickness of central subfield | 41.89 ± 8.81 | 40.56 ± 9.14 | 40.09 ± 8.90 | 40.27 ± 8.95 | **<0.005** | **<0.005** | **<0.005** |
| ISOS-RPE thickness of inner subfield | 37.92 ± 7.07 | 37.30 ± 7.49 | 36.92 ± 7.42 | 37.15 ± 7.25 | 0.109 | **0.01** | **0.048** |
| ISOS-RPE thickness of outer subfield | 36.96 ± 6.10 | 36.25 ± 6.71 | 35.63 ± 6.89 | 36.14 ± 6.30 | **0.03** | **<0.005** | **0.014** |
| Macular thickness at the central subfield | 268.23 ± 31.69 | 272.69 ± 30.73 | 272.67 ± 34.92 | 269.49 ± 36.27 | **<0.005** | **0.012** | 0.491 |
| Macular thickness at the inner inferior subfield | 309.00 ± 27.47 | 306.09 ± 26.01 | 306.18 ± 32.32 | 304.11 ± 31.07 | **0.038** | 0.079 | **<0.005** |
| Macular thickness at the inner nasal subfield | 315.09 ± 26.69 | 311.95 ± 26.94 | 311.13 ± 29.22 | 309.25 ± 35.07 | **0.025** | **<0.005** | **<0.005** |
| Macular thickness at the inner superior subfield | 311.19 ± 30.63 | 306.39 ± 27.85 | 306.12 ± 29.61 | 304.21 ± 34.92 | **<0.005** | **<0.005** | **<0.005** |
| Macular thickness at the inner temporal subfield | 301.37 ± 26.65 | 298.33 ± 25.87 | 298.82 ± 26.68 | 296.68 ± 31.23 | **0.028** | 0.073 | **<0.005** |
| Macular thickness at the outer inferior subfield | 263.09 ± 24.30 | 261.39 ± 27.51 | 261.71 ± 32.68 | 260.12 ± 25.89 | 0.215 | 0.370 | **0.028** |
| Macular thickness at the outer nasal subfield | 283.10 ± 22.13 | 279.61 ± 23.71 | 278.78 ± 29.10 | 277.19 ± 26.89 | **<0.005** | **<0.005** | **<0.005** |
| Macular thickness at the outer superior subfield | 269.34 ± 22.96 | 265.83 ± 25.96 | 266.00 ± 26.17 | 264.33 ± 25.98 | **<0.005** | **0.011** | **<0.005** |
| Macular thickness at the outer temporal subfield | 258.97 ± 22.95 | 257.09 ± 22.63 | 258.47 ± 23.07 | 256.54 ± 23.24 | 0.115 | 0.681 | 0.05 |
| Overall macular thickness | 277.62 ± 20.82 | 274.98 ± 19.75 | 275.16 ± 24.11 | 273.35 ± 23.35 | **0.013** | **0.04** | **<0.005** |
| IOP Corneal compensated | 16.04 ± 4.24 | 16.28 ± 5.28 | 16.17 ± 4.96 | 16.25 ± 5.72 | 0.329 | 0.573 | 0.428 |
| IOP Goldmann correlated | 15.90 ± 4.08 | 15.90 ± 4.21 | 15.98 ± 4.24 | 15.92 ± 4.27 | 0.996 | 0.735 | 0.924 |

*Significant p values are represented in bold.*
